# ComK-induced cell death is reversed by upregulating the SigB or Spx pathway in *Bacillus subtilis*

**DOI:** 10.1128/spectrum.01612-25

**Published:** 2025-08-07

**Authors:** Emma E. Wiesler, Qin Liao, Zhongqing Ren, Kathy F. Zhang, Jin Dai, Yinuo Ma, Gail G. Hardy, Xindan Wang

**Affiliations:** 1 Department of Biology, Indiana University 1772, Bloomington, Indiana, USA; University of Manitoba, Winnipeg, Manitoba, Canada

**Keywords:** natural competence, ComK, Spx, SigB, replication initiation, cell division, *Bacillus subtilis*

## Abstract

Natural competence of bacteria is an important tool for researchers to rapidly engineer genetic mutations. In *Bacillus subtilis*, the master regulator for competence is ComK*,* which is typically only expressed during stationary phase, allowing cells to uptake DNA and incorporate it into the genome. Here, we engineered cells to overexpress *comK* in exponential phase using inducible promoters, thereby inducing competence without going through stationary phase. We observed that overexpressing *comK* in exponentially growing cells inhibited DNA replication and cell division, leading to low DNA content, elongated cells, and eventually cell death. To rescue *comK*-induced cell death, we isolated suppressors of *comK* overexpression using both spontaneous suppression and a transposon-based method (transposon sequencing). From both methods, the suppressors upregulated either the sigma B (SigB) or Spx pathway, which are known to be activated in response to stress. Importantly, while upregulating SigB or Spx suppressed cell death, it also reduced competence by lowering ComK protein levels. Altogether, our results collectively show that during competence, normal cellular functions such as DNA replication and cell division are inhibited. While cells can upregulate stress responses to facilitate these normal cellular functions, these processes inadvertently lower ComK levels and inhibit competence. Our study highlights the sophisticated cellular regulatory systems that specifically direct cell growth, competence, or stress management.

**IMPORTANCE:**

Naturally competent bacteria, such as *Bacillus subtilis,* take up DNA to use it as nutrients and generate new genotypes. Researchers exploit natural competence to manipulate bacterial genomes. While all *B. subtilis* cells in a population are capable of being competent, only a small portion of cells do so. To synchronously induce competence in a cell population, we overexpressed the master competence regulator *comK*. We found that competence is associated with inhibited cellular functions, such as cell division and DNA replication. Suppressor mutant screens revealed that increasing expression of either stress regulator sigma B or Spx restores cell division and DNA replication functions, but in turn inhibits competence. Our study emphasizes the complex regulatory networks that balance cell growth, competence, and stress responses.

## INTRODUCTION

Natural competence is a useful tool for researchers to manipulate bacterial genotypes. New strains can be used not only to study bacterial cellular functions and biological processes, but also to produce chemicals and enzymes for industrial use (1–3). *Bacillus subtilis* is an important naturally competent model bacterium and industrial workhorse. Compared with other bacterial species that are naturally competent, *B. subtilis* has a lower transformation efficiency. For instance, *Streptococcus pneumoniae* and *Vibrio cholerae* can have a transformation efficiency of ~1/10, which is roughly 1,000-fold higher than what has been achieved in *B. subtilis* (4, 5). High frequency of transformation allows researchers to develop methods like MuGENT to multiplex and introduce several mutations into a strain in a single transformation (5–7). MuGENT has not yet been accomplished in *B. subtilis*, which motivated us to find ways to increase the transformation frequency in this bacterium.

*B. subtilis* competence is growth phase dependent. When cells enter the stationary phase, a small subset of cells (~10%) becomes competent (8–12). Competence development in *B. subtilis* is controlled by the master regulator ComK, which is under complex regulation (8, 13–16). At the transcriptional level, *comK* is regulated by at least six proteins that act as repressors or activators (reviewed in reference 16). Post-translationally, ComK protein levels are tightly controlled (17–22). ComK binds to the *comK* promoter and positively regulates its own expression (14). During the stationary phase, this complex regulation leads to a state called bistability in which ~10% of the cells are competent while the majority of the population is not (10, 23–25).

To increase the fraction of competent cells in the cell population, previous studies tried to bypass the regulations of *comK* (26). However, this resulted in cell death and nucleoid separation defects (26). We reasoned that if we could suppress ComK-induced cell death and allow cells to be healthy and thrive, we could increase transformation efficiency in *B. subtilis* and facilitate the ability to generate multiple mutations at the same time like in other model organisms (5–7). Driven by this straightforward idea, we sought to isolate suppressors in ComK-overexpressing cells that reversed the cell death phenotype.

## RESULTS

### Ectopic expression of *ComK* increases competence

**Fig 1:**
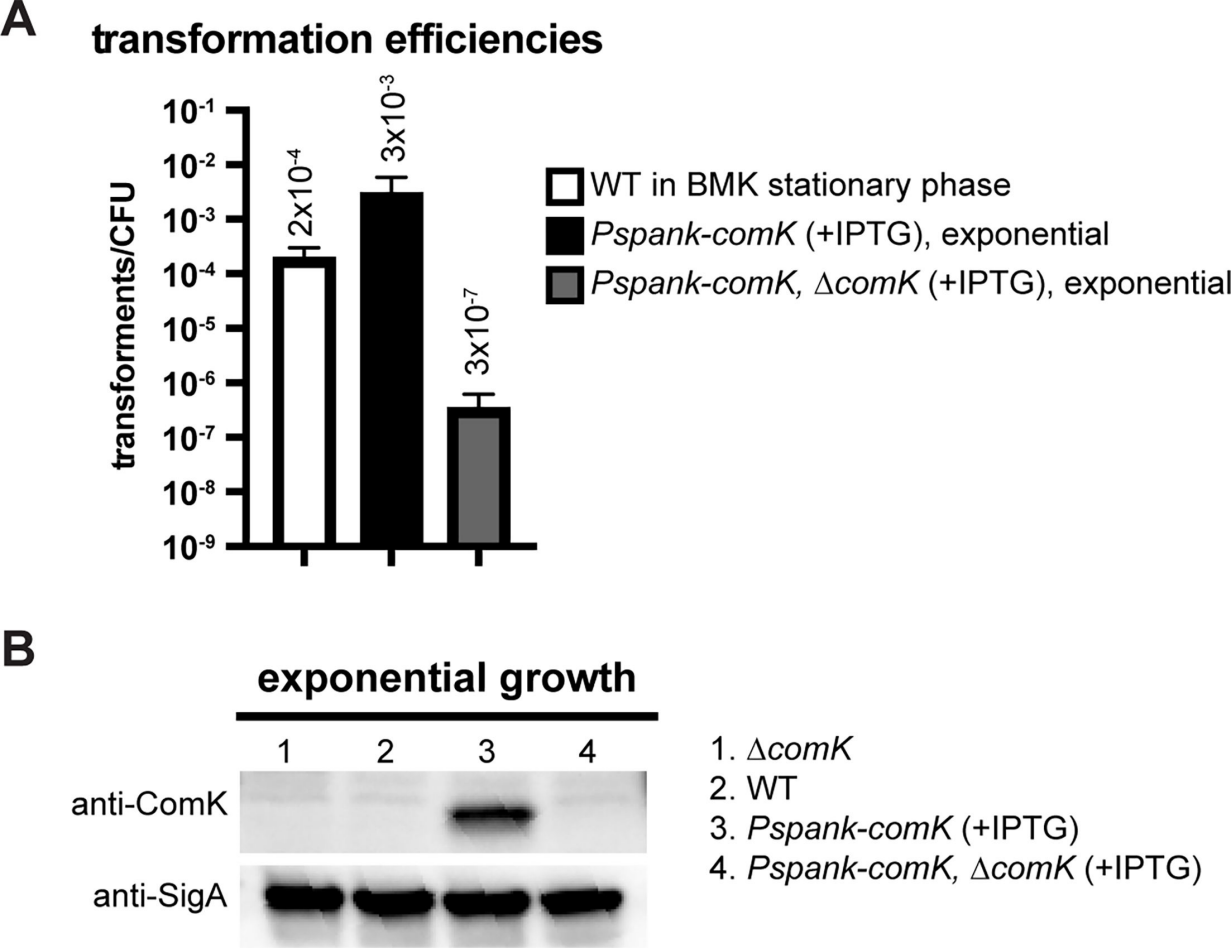
Ectopic expression of *comK* increases competence. (**A**) Transformation efficiencies of indicated strains: wild-type PY79 (WT) growing in BMK media, and BWX4497 (*Pspank-comK*) and BWX3253 (*Pspank-comK*, *∆comK*) growing exponentially in casein hydrolysate (CH) media containing 1 mM IPTG. At least three biological replicates consisting of three technical replicates were performed. Error bars show standard deviations. (**B**) Immunoblot analysis using anti-ComK in indicated strains: BWX3145 (*∆comK*), WT (PY79), BWX4497 (*Pspank-comK*), and BWX3253 (*Pspank-comK*, *∆comK*) growing exponentially in CH media containing 1 mM IPTG. Anti-SigA was used as a loading control.

To determine ComK levels during standard competence procedure, cells were grown in Byoung-Mo Koo (BMK) competence medium (27) for 4 hours to reach stationary phase and become competent. Using a PCR product, the transformation efficiency (i.e., number of transformants per colony-forming unit [CFU]) was about 2 × 10^−4^ (Fig. 1A). To synchronously induce competence in an entire cell population instead of just a portion during stationary growth (10, 23–25), we created a *comK* overexpression strain using an Isopropyl β-D-thiogalactopyranoside (IPTG)-inducible promoter (*Pspank*) (28). In the presence of IPTG*,* the transformation efficiency was increased by 10-fold (Fig. 1A). Interestingly, deleting the endogenous copy of *comK* reduced competence by ~10,000-fold in *Pspank-comK* (to 3 × 10^−7^) (Fig. 1A). Knowing that ComK protein levels are tightly controlled (17–22) and ComK positively regulates its own expression (14), we examined ComK protein levels using immunoblot analysis. We found that when *comK* was induced by *Pspank,* the ComK protein only accumulated when the native copy of *comK* was present (Fig. 1B). Our results show that even when *comK* is ectopically expressed, the autoregulation of *comK* is required for ComK protein accumulation and cell competence (Fig. 1A and B).

### ComK caused cell death by inhibiting cell division and DNA replication

**Fig 2:**
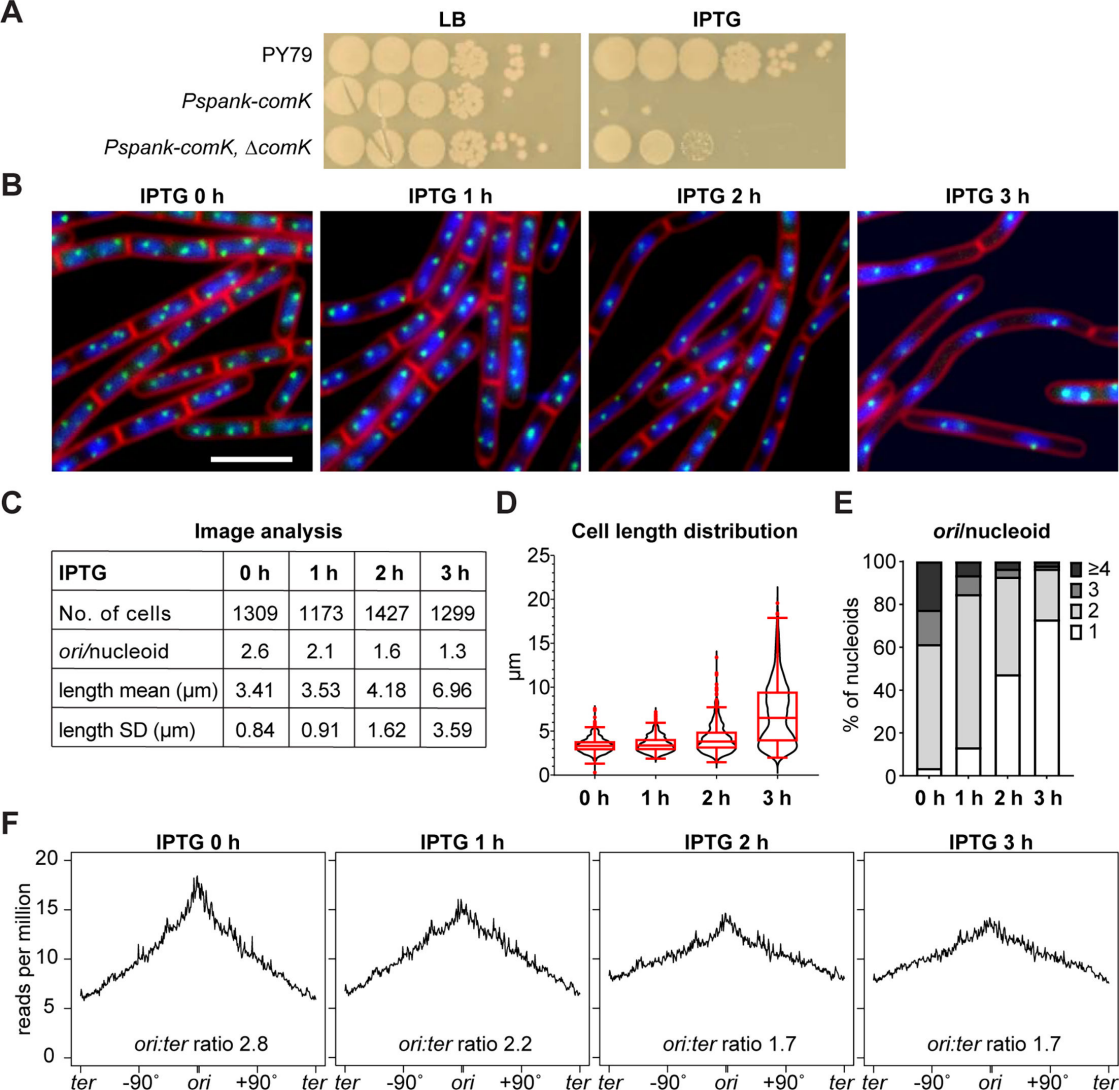
*comK* causes cell death by inhibiting cell division and DNA replication. (**A**) Serial dilutions of wild-type PY79 (WT), a *comK*-expressing strain (BWX4516), and a *comK*-expressing strain *∆comK* (BWX3253). *comK* was expressed from an IPTG-inducible promoter (*Pspank*). Cells were grown in Luria broth (LB) medium and normalized to an OD_600_ of 2. The cultures were serially diluted and spotted on LB agar plates with or without 0.5 mM IPTG. (**B**) *comK*-expressing strain (BWX4516) was grown in casein hydrolysate medium in the absence or presence of 1 mM IPTG for the indicated times. 4´,6-Diamidino-2-phenylindole-stained nucleoids (blue), FM4-64-stained membrane (red), and replication origin (*tetO*/TetR-CFP, green) are shown. Bar, 4 µm. (**C**) Analysis of the average number of origins per nucleoid and the mean length of cells shown in (**B**). (**D**) Quantitative analysis of cell length distribution of cells shown in (**B**). Boxplots show the mean, quartiles, and 5th and 95th percentile of the data. (**E**) Quantitative analysis of the number of origins per nucleoid of cells in (**B**). (**F**) Marker frequency plots of BWX3174 cells in the absence or presence of *comK* expression at indicated time points. The *x*-axis represents genome coordinates. The *y*-axis represents the number of reads which were normalized to the total reads (see details in Materials and Methods). The copy number of the origin relative to the terminus is shown.

Although induction of *Pspank-comK* increased transformation efficiency (Fig. 1A), we found that this condition abolished cell viability (Fig. 2A). This is consistent with previous observations using cells in native competent state (26). In line with the ComK protein levels (Fig. 1B), the lethal effect of *Pspank-comK* also requires the native copy of *comK*. To understand the lethal effects of *comK* overexpression at the cellular level, we examined cell length and replication status using fluorescence microscopy upon *comK* expression (Fig. 2B). We visualized the nucleoid and cell membrane by staining, as well as replication origins using *tetO48* array bound by the *tet* repressor fused with cyan fluorescent protein (TetR-CFP) (29). We found that cells were elongated gradually after induction of *comK* expression. After 3 hours of *comK* expression, the average cell length increased from 3.41 µm to 6.96 µm (Fig. 2B through D). In addition, the gaps between the nucleoids increased, and the number of origins per nucleoid decreased from an average of 2.6 before induction to 1.3 per nucleoid after 3 hours of induction (Fig. 2B, C, and E). Genome-wide marker frequency analysis using whole genome sequencing (WGS) suggests that the ratio of *ori* relative to *ter* decreased from 2.8 to 1.7 (Fig. 2F). These data suggest that *comK* overexpression during exponential growth inhibits both cell division and DNA replication initiation, eventually leading to cell death (Fig. 2A), consistent with previous observations (26, 30).

### Selection of suppressors that rescue ComK-induced cell death

**Fig 3:**
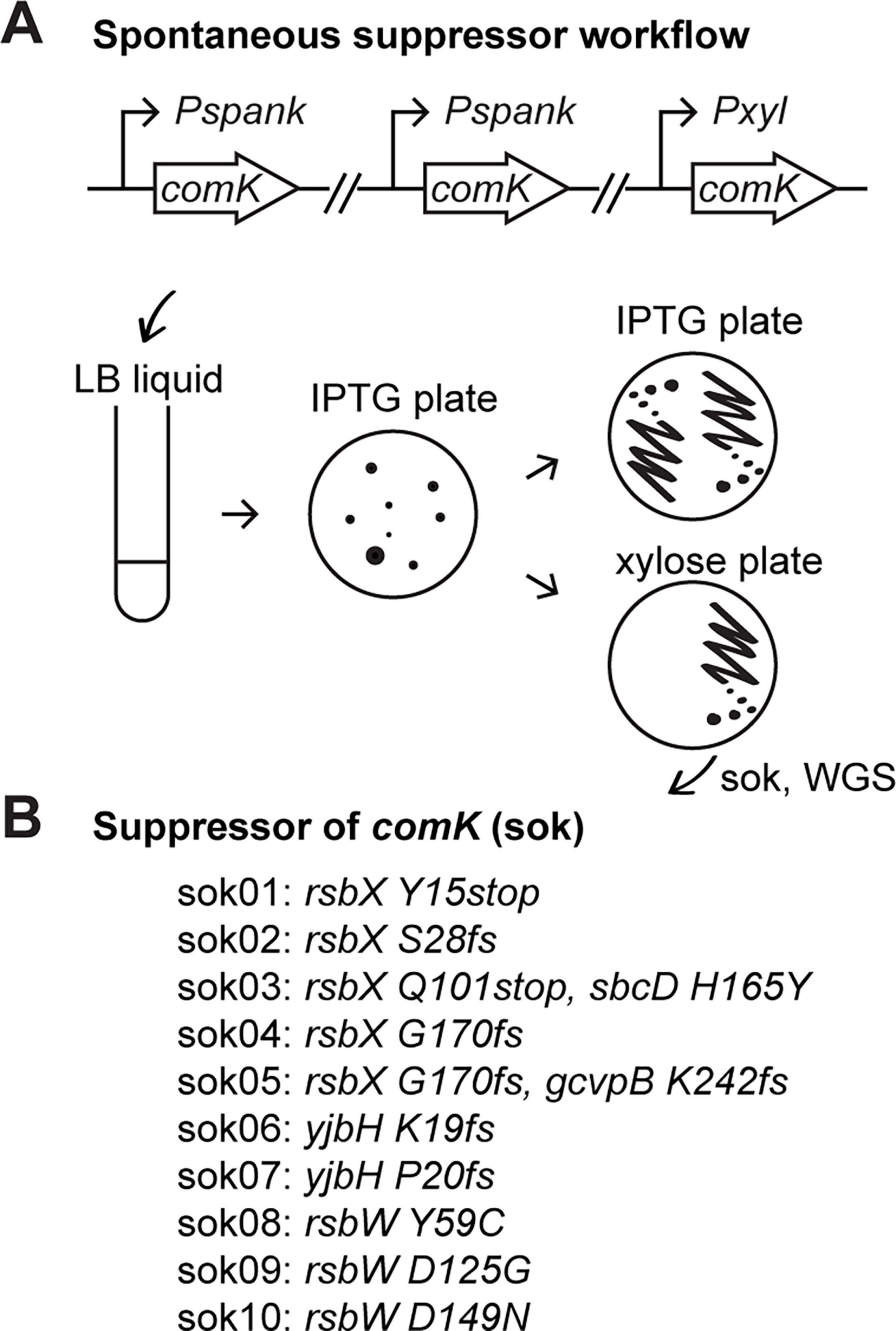
Spontaneous mutations that suppress *comK*-induced cell death. (**A**) The workflow for the selection of spontaneous suppressors in a *comK*-expressing strain (BWX4518), which has two copies of IPTG-inducible (*Pspank*) *comK* and one copy of xylose-inducible (*Pxyl*) *comK*. Cells were grown in Luria broth (LB) liquid medium, then spread on plates containing 0.5 mM IPTG. Colonies were streaked on IPTG and xylose plates. Only the colonies that grew on both plates were processed for whole genome sequencing to identify soks. The details of the selection screen can be found in Materials and Methods. (**B**) The list of soks identified from the screen. Mutations in soks are stated. “fs” means frameshift.

To bypass ComK-induced cell death, we isolated spontaneous suppressors in a *comK*-expressing strain. This strain contained two copies of IPTG-inducible *comK* inserted at different genetic loci to reduce the false-positive hits that reside in the *lacO/lacI* expression control system*,* and one copy of xylose-inducible *comK* for validation purposes described below (Fig. 3A). In addition, we kept the native copy of *comK* which is required for competence (Fig. 1A) and cell death (Fig. 2A). The cells were grown in liquid culture without inducers, then spread on plates containing 0.5 mM IPTG. The colonies that grew on IPTG plates were candidate suppressors that rescued ComK-induced cell death. The frequency of suppression was about 10^−5^. The candidates were further streaked on IPTG plates as well as xylose plates. The isolates that failed to grow on xylose were not true suppressors but likely contained mutations in the *lacO/lacI* control system and were omitted from further testing. Those that grew on xylose plates were candidate suppressors of ComK (soks)-induced cell death and were further analyzed by WGS. To avoid picking siblings, this experiment was performed in 10 independent liquid cultures, and only one suppressor isolate per culture was chosen for sequencing. We identified 10 soks: two had frameshift mutations in *yjbH*, five had nonsense or frameshift mutations in *rsbX*, and three had missense mutations in *rsbW,* specifically *rsbW* (Y59C), *rsbW* (D125G), and *rsbW* (D149N) (Fig. 3B).

**Fig 4:**
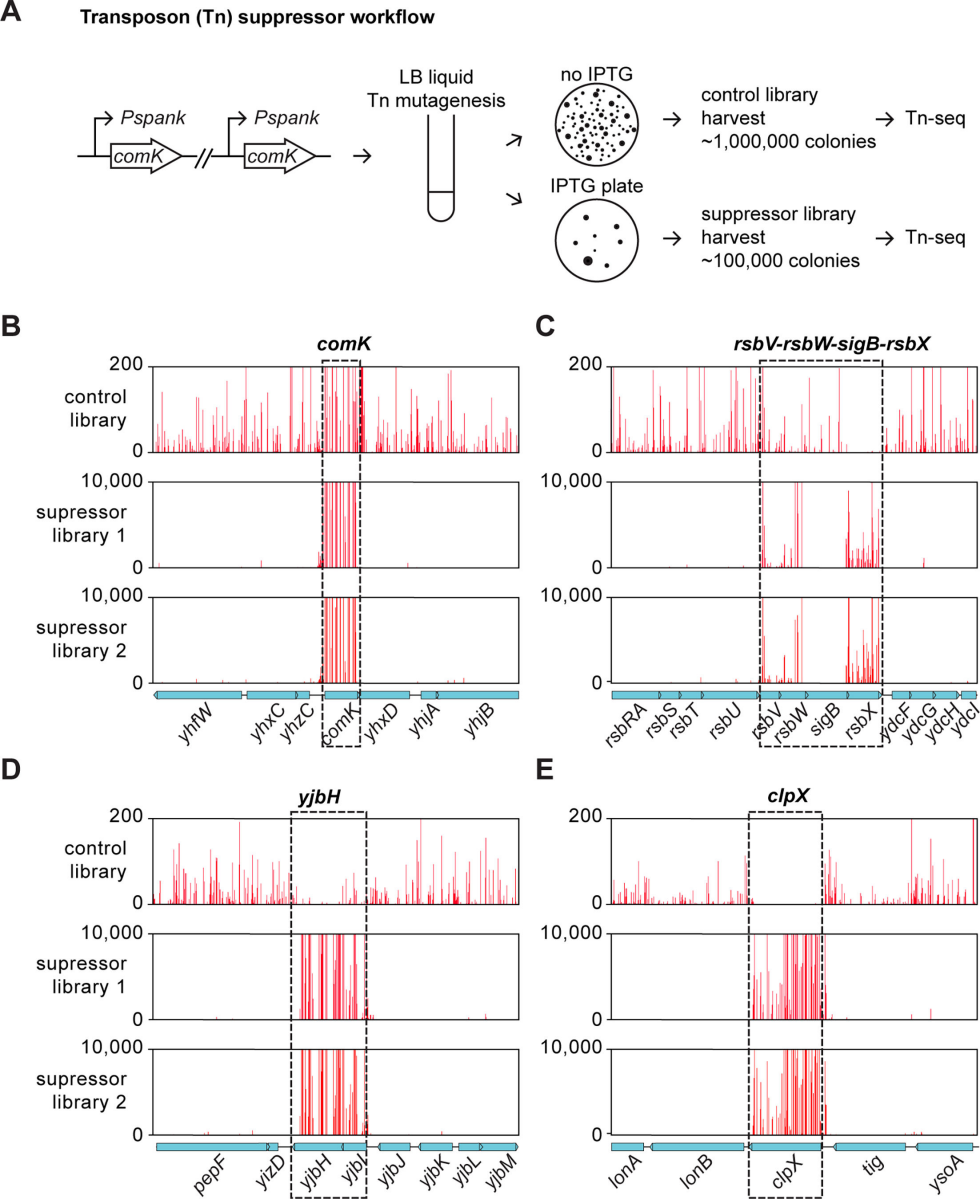
Transposon insertions that suppress *comK*-induced cell death. (**A**) The workflow to identify transposon insertions that suppress *comK*-induced cell death in a *comK*-expressing strain (BWX4512), which has two copies of IPTG-inducible (*Pspank*) *comK*. The transposon-carrying plasmid (pWX642) was transformed into BWX4512. The transformants were grown in Luria broth (LB) liquid medium with spectinomycin to allow mutagenesis. Transposants were spread on spectinomycin plates containing 0.5 mM IPTG for suppressors or without IPTG for control. Colonies were scraped and processed for Tn-seq. The details can be found in Materials and Methods. (**B through E**) Tn-seq plots showing transposon insertions in the absence of *comK* expression (the control, top) or in the presence of *comK* expression (suppressor selection, bottom, two biological replicates). The *x*-axis indicates gene locus. The *y*-axis indicates the number of sequencing reads at each insertion site. Black dotted rectangles highlight regions of interest.

Our spontaneous suppressor screening identified 10 soks and captured different types of mutations. However, this approach was limited by sampling depth. Next, we performed a transposon-sequencing (Tn-seq) screen as a complementary approach to systematically identify potential soks (Fig. 4A). We introduced a transposon-containing plasmid (pWX642) (31) into BWX4512, which has two copies of IPTG-inducible *comK,* to reduce the chance of a transposon disrupting the induction system (Fig. 4A). After transposon mutagenesis, cells were plated on plates without or with IPTG for control or suppressor selection, respectively. Two biological replicates of transposon libraries were harvested for each condition. For each biological replicate, about 1 × 10^6^ colonies were harvested from the control library and 1 × 10^5^ colonies from the suppressor selection library. The suppressor frequency was estimated to be ~10^−4^. We extracted genomic DNA from each library and performed Tn-seq as described previously (31). For the control libraries, we observed transposon insertions in non-essential genes in a similar profile as seen previously (32). For the suppressor libraries, we found that the transposon insertions were only in a few genes (Fig. 4B through E). First, we observed transposon insertions at the native *comK* gene locus (Fig. 4B). Since transposon insertions in all three copies of *comK* (one endogenous copy and two ectopic copies) were mapped to the native *comK* locus, we reasoned that these insertions abolished the expression of *comK* either from the ectopic expression systems or from the native locus which is needed for cell death to occur (Fig. 2A and 4A). Next, we found high insertion counts in *yjbH*, *rsbX*, and *rsbW* in the suppresser libraries (Fig. 4C and D), suggesting their potential roles in rescuing ComK-induced cell death. This is consistent with our earlier spontaneous suppressors screen (Fig. 3). Finally, we identified three new suppressor candidates, *rsbV* (Fig. 4C), *yjbI* (Fig. 4D)*,* and *clpX* (Fig. 4E), which did not appear in our spontaneous suppressor screen.

### Validation of suppressors

**Fig 5:**
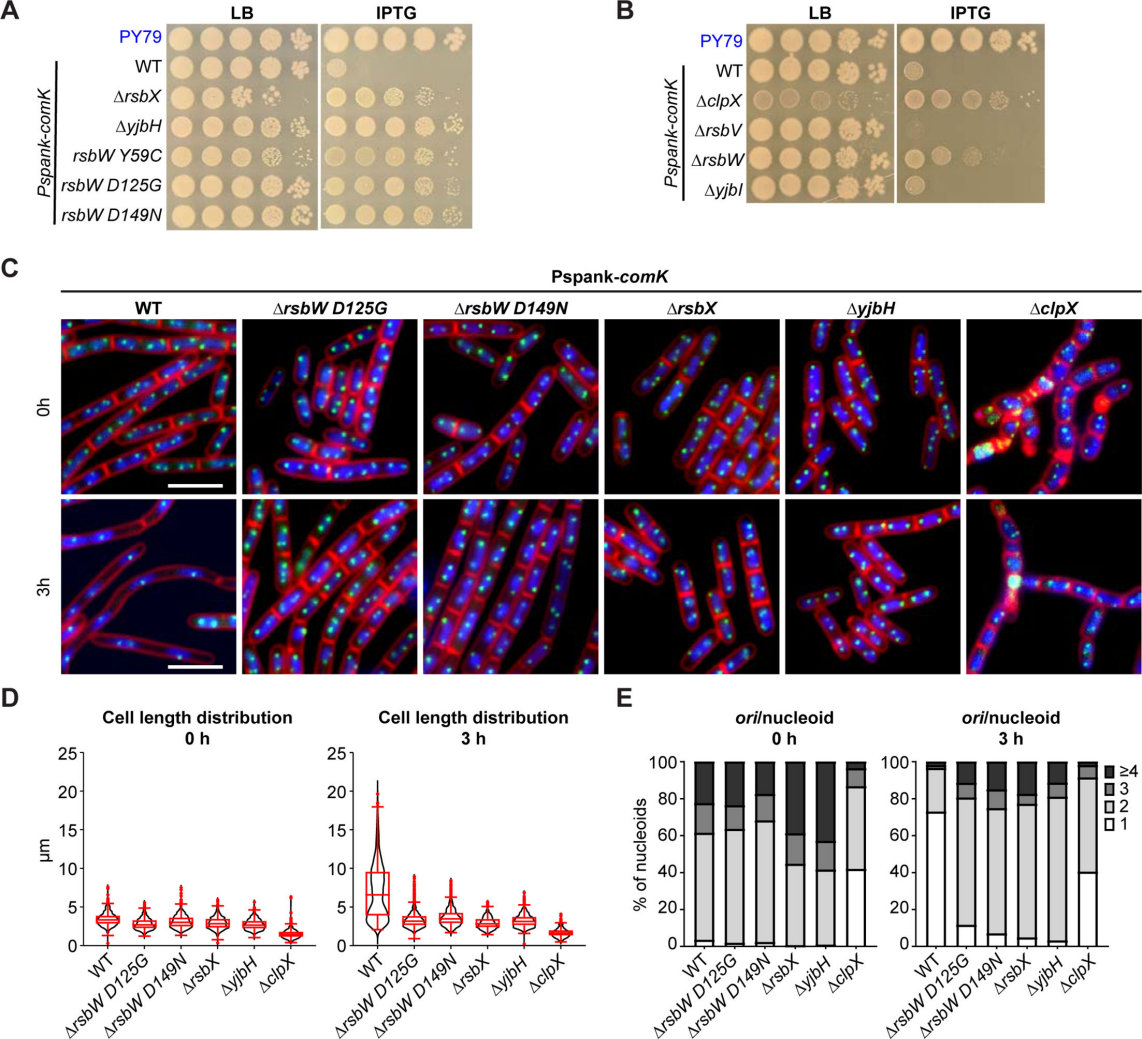
Validation of the suppressors. (**A**) Serial dilutions of indicated strains. Cells were grown in Luria broth (LB) medium and normalized to an OD_600_ of 2. The cultures were serially diluted and spotted on agar plates with or without 0.5 mM IPTG. The top strain was the true wild-type PY79 (WT) strain. The six strains below all contained IPTG-inducible (*Pspank*) *comK*. Strains from top to the bottom: PY79, BWX4661, BWX4699, BWX4700, BWX4759, BWX4760, BWX4761. (**B**) Serial dilutions of indicated strains processed the same way as in (**A**). The top strain was the true WT PY79 strain. The five strains below all contained IPTG-inducible (*Pspank*) *comK*. Strains from the top to the bottom: PY79, BWX4661, BWX4825, BWX5627, BWX5620, BWX5618. (**C**) The suppressors rescued ComK’s inhibition of cell division and DNA replication. Representative micrographs of indicated strains (BWX4516, BWX4695, BWX4696, BWX4766, BWX4767, BWX4827). Cells were grown in casein hydrolysate medium (top panels), then 1 mM IPTG was added to induce *comK* expression for 3 hours (bottom panels). 4´,6-Diamidino-2-phenylindole-stained nucleoids (blue), FM4-64-stained membrane (red), and replication origin (*tetO*/TetR-CFP, green) are shown. Bar, 4 µm. The images of WT have been shown in Fig. 2B. (**D**) Cell length distribution of cells in (**C**). At least 157 cells were analyzed for each condition. Boxplots show the mean, quartiles, and 5th and 95th percentile of the data. (**E**) Number of origins per nucleoid of cells in (**C**). At least 632 cells were analyzed for each condition.

To validate whether the hits from both screens were true suppressors, we made in-frame deletions of *rsbV*, *rsbW*, *rsbX, yjbI, yjbH,* and *clpX,* as well as the three-point mutations *rbsW* (Y59C), *rbsW* (D125G), and *rbsW* (D149N), in a strain containing *Pspank-comK*. As shown earlier, IPTG-inducible expression of *comK* severely impaired the cell growth (Fig. 2A, 5A and B). However, cell viability was restored in the mutants of *yjbH, clpX, rsbX,* and *rsbW* (Fig. 5A and B), demonstrating that the deletions and point mutations of these genes suppressed ComK-induced cell death.

Notably, although transposon insertions in *rsbV* and *yjbI* showed up as suppressors in our Tn-seq experiment (Fig. 4C and D), in-frame deletions of these genes failed to restore cell growth upon *comK* expression (Fig. 5B). Thus, ∆*rsbV* and ∆*yjbI* are not true suppressors. Since *rsbV* and *yjbI* are immediately upstream of validated suppressors *rsbW* and *yjbH*, respectively, we posited that the transposon insertions in *rsbV* and *yjbI* had polar effects and were also disrupting downstream genes, which caused suppression of ComK-induced death. (Fig. 4C and D).

To understand how the suppressors rescue ComK-induced cell death, we combined the suppressor mutations (∆*rsbX*, ∆*yjbH,* or ∆*clpX*) with *comK* expression and analyzed cell division and DNA replication using fluorescence microscopy (Fig. 5C). Of the four *rsbW* mutations, *rsbW* (D125G) and *rsbW* (D149N) exhibited better growth than *rsbW* (Y59C) and ∆*rsbW* (Fig. 5A and B). Thus, we proceeded with *rsbW* (D125G) and *rsbW* (D149N) for microscopy analysis. As shown in Fig. 2, after 3 hours of *comK* expression in otherwise wild-type cells, cell division and replication initiation were inhibited. However, in the presence of suppressor mutations, *rsbW* (D125G), *rsbW* (D149N), ∆*rsbX*, ∆*yjbH,* or ∆*clpX,* cell division and replication occurred (Fig. 5C throuogh E). Thus, these suppressor mutants allowed cell division and DNA replication to occur even when *comK* expression was induced.

### Upregulation of sigma B (SigB) or Spx rescues ComK-induced death

According to the known functions of their gene products, our suppressor mutations can be divided into two groups. In the first group, RsbW and RsbX are negative regulators of the activity of SigB, which is an alternative sigma factor that upregulates ~150 genes when cells are under cellular or environmental stress (33–39). Specifically, RsbW, acting as an anti-sigma factor of SigB, binds to SigB and prevents SigB from interacting with RNA polymerase to express the SigB regulon (35–37). RsbX acts in a regulatory cascade that ultimately results in the upregulation of SigB (40). In light of this knowledge, we posited that the suppressors (∆*rsbX*, ∆*rsbW*, *rsbW* (D125G), *rsbW* (D149N), and *rsbW* (Y59C) identified in our screens likely upregulate the SigB regulon. In the second group, *yjbH* and *clpX* are involved in negatively regulating the level of the Spx protein, which is a global regulator that functions in stress response (20, 41). YjbH binds to Spx and facilitates the degradation of Spx by the ClpXP protease complex (42). Therefore, the disruption of *yjbH* or *clpX* results in higher levels of Spx and upregulation of its regulon. Based on these existing insights, we hypothesized that a higher level of SigB or Spx can suppress ComK-induced cell death.

**Fig 6:**
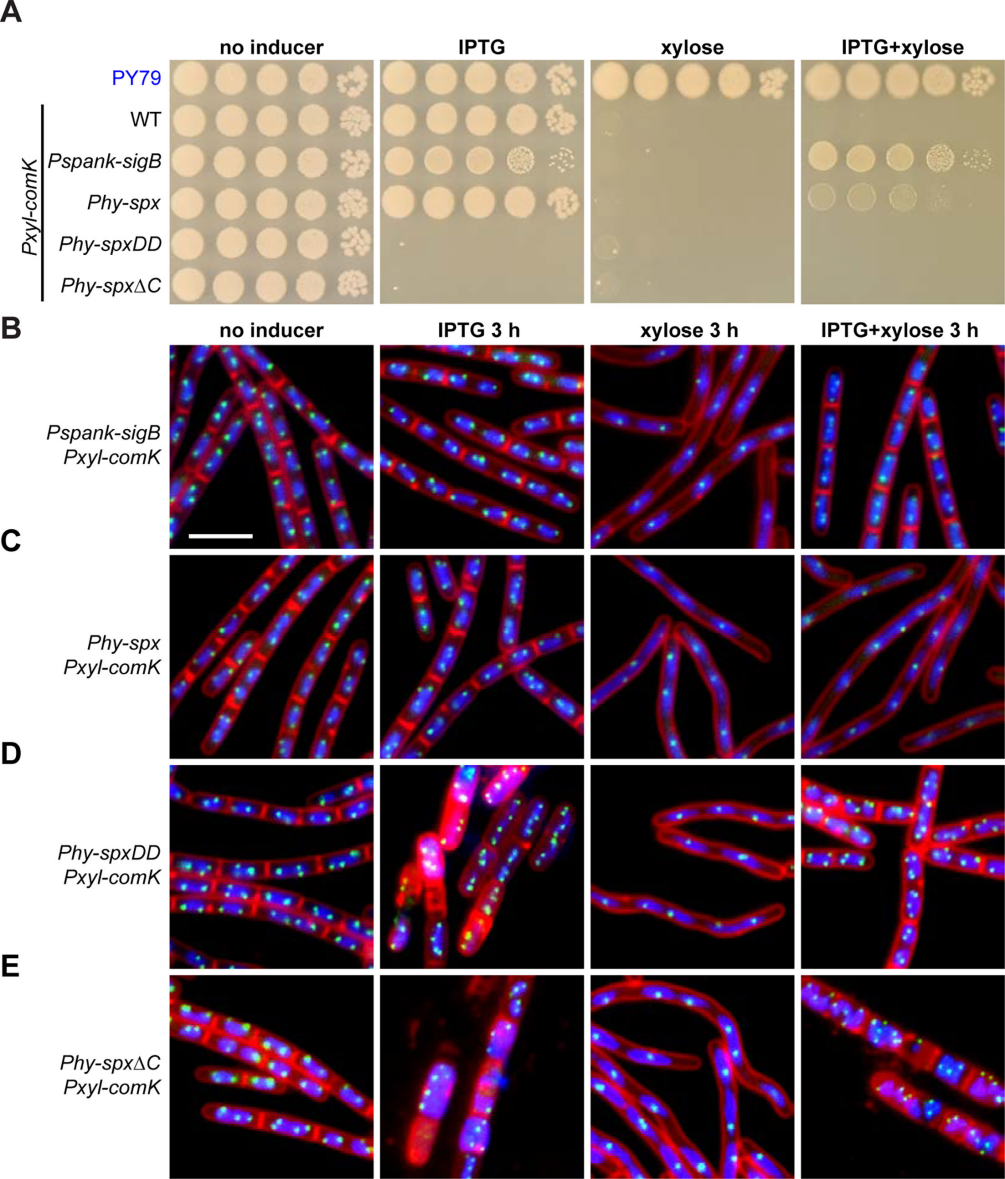
Expression of SigB or Spx rescues *comK*-induced cell death. (**A**) Serial dilutions of indicated strains on Luria broth (LB) agar plates with or without 0.5 mM IPTG and/or 0.5% xylose as indicated. The top strain was the true wild-type PY79 (WT) strain. The seven strains below all contained xylose-inducible *comK*. Strains from the top to the bottom: PY79, BWX4788, BWX4809, BWX4868, BWX4866, BWX4867. (**B through E**) Representative micrographs of indicated strains (BWX4809, BWX4868, BWX4866, BWX4867). Cultures were grown with or without the indicated inducers, 1 mM IPTG and/or 0.5% xylose. 4´,6-Diamidino-2-phenylindole-stained nucleoids (blue), FM4-64-stained membrane (red), and replication origin (*tetO*/TetR-CFP, green) are shown. Bar, 4 µm. Quantitative analysis can be found in Fig. S1.

To test this hypothesis, we constructed strains that contained a xylose-inducible *comK* and an IPTG-inducible *sigB* or *spx*. As seen in Fig. 6A (IPTG+xylose), overexpression of *sigB* rescued cell death, supporting our hypothesis. However, overexpression of the wild-type *spx* allele had very modest suppression. Given that the Spx protein is subjected to degradation by ClpXP (18, 43, 44), we questioned whether expressing non-degradable alleles of Spx would rescue the ComK-induced death. It has been shown that mutating the two aspartic acid residues (i.e., the *spxDD* allele) or deleting the 12 amino acids at the C-terminus of Spx (i.e., the *spx*∆*C* allele) renders the mutants non-degradable (45, 46). Unfortunately, we observed that expression of these two non-degradable alleles resulted in cell death on their own (Fig. 6A, IPTG). Therefore, we could not determine the role of these *spx* alleles in the suppression of ComK-induced death using this plating assay.

Next, we investigated the effect of *sigB* and *spx* expression on the cytological behavior of *comK*-expressing cells using fluorescence microscopy (Fig. 6B and C). After 3 hours of *sigB* induction alone, there was little effect on cell length or DNA replication (Fig. 6B and S1A). When *comK* was overexpressed alone, there was inhibition of cell division and DNA replication as observed above (Fig. 2 and 6B and S1A). Finally, when *sigB* and *comK* were overexpressed, cell division and cell growth were restored to close to wild-type levels (Fig. 6B and S1A). These results indicate that expression of *sigB* allows cell division and DNA replication even when *comK* expression was induced.

When *spx* alone was overexpressed, cell growth or cell division was barely impacted (Fig. 6C and  Fig. S1B). When *comK* was also overexpressed, the presence of Spx did not rescue cell division or DNA replication (Fig. 6C and Fig. S1B). These results are consistent with the mild rescue effect on our plating assay (Fig. 6A) and with the idea that Spx is subjected to degradation and might not accumulate to high enough levels for suppression. Therefore, we tested the cytological effect of the two non-degradable Spx variants (SpxDD and Spx∆C) (45, 46). Consistent with cell death observed in our plating assay (Fig. 6A), the expression of *spxDD* or *spx*∆*C* resulted in extremely sick cells (Fig. 6D and E; IPTG panel). We found that SpxDD and Spx∆C led to increased origin numbers per nucleoid, suggesting over-replication of DNA (Fig. S1C and D). Nonetheless, their expression rescued inhibition of cell division and DNA replication seen in *comK* overexpression (Fig. 6D and E; Fig. S1C and D).

These data support the hypothesis that increased levels of SigB or Spx allow cell division, DNA replication, and cell survival even when *comK* expression is induced.

### Rescuing ComK-induced death reduces competence

Our motivation for this study was to induce competence synchronously in an exponentially growing culture to increase transformation frequency and aid strain engineering. Based on growth phenotypes (Fig. 5A and 6A) and the mechanism of suppression, we chose three of our best-performing suppressors to proceed: *rsbW* (D149N), *sigB* overexpression (*sigB++*)*,* and ∆*yjbH* which increases Spx levels (42).

**Fig 7:**
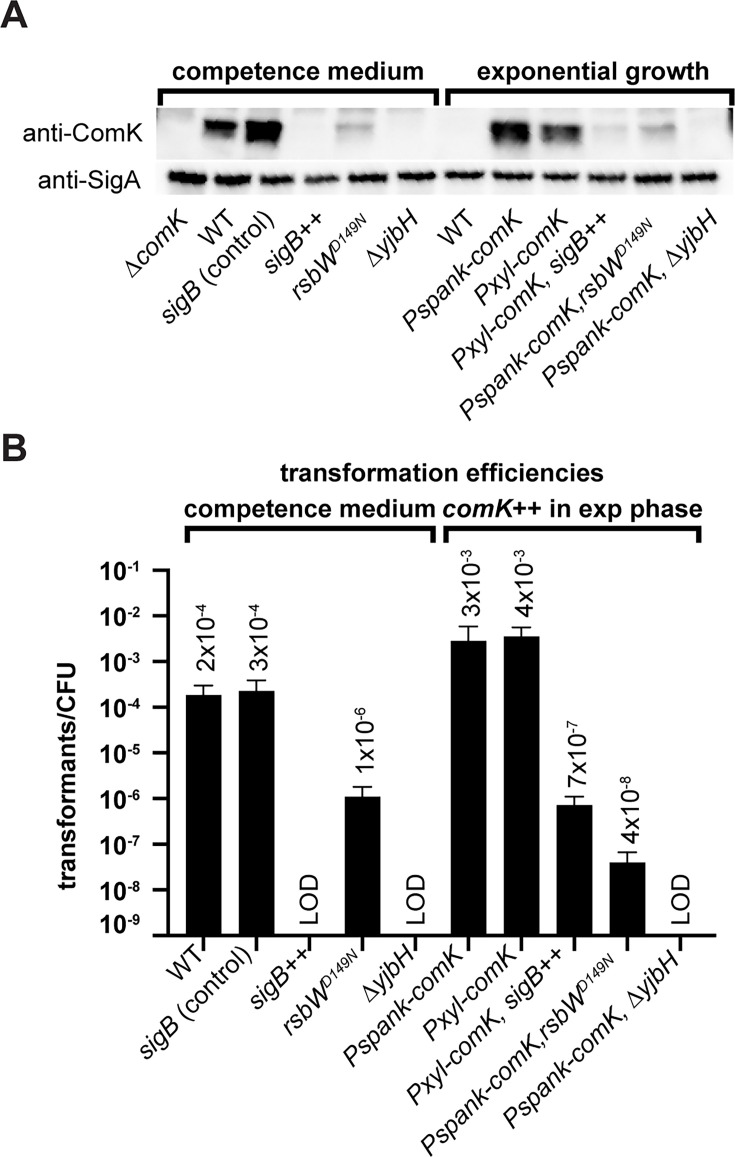
The suppressors reduced ComK protein levels and abolished competence. (**A**) Immunoblot analysis using anti-ComK in indicated strains from left to right: BWX3145, PY79, BWX4795, BWX4795, BWX4751, BWX4643, PY79, BWX4497, BWX4513, BWX5716, BWX4755, and BWX4696. Anti-SigA was used as a loading control. The first five strains were grown in BMK competence medium. The last five strains were grown in casein hydrolysate (CH) medium containing the appropriate inducer (1 mM IPTG or 0.5% xylose). (**B**) Cells were transformed with 5 ng of PCR product containing a spectinomycin antibiotic marker flanked with 2–3 kb of homology upstream and downstream of the *hbs* gene locus. Strains from left to right: PY79, BWX4795, BWX4795, BWX4751, BWX4643, BWX4497, BWX4513, BWX5716, BWX4755, and BWX4696. The first five strains were grown in BMK competence medium. The last five strains were grown in CH medium containing the appropriate inducer (1 mM IPTG or 0.5% xylose). Transformation efficiency was calculated by dividing the number of transformants (CFUs on spectinomycin plates) by the total number of colonies (CFUs on Luria broth (LB) plates). At least three biological replicates were performed. Each biological replicate contains three technical replicates that were averaged to calculate transformation efficiency. Error bars show standard deviations. See details in Materials and Methods.

A recent study showed that SigB induces the expression of antisense RNA of *comK* which inhibits competence (47). Since the antisense RNA inhibits *comK* translation both *in cis* and *in trans* (47), it is possible that the same mechanism would counteract the overexpression of ComK in our system, thereby rescuing cell death simply by eliminating ComK, the root cause of cell death. Similarly, Spx is a transcriptional regulator that enables cells to withstand a wide range of stressors (48, 49). *In vitro* evidence indicates that Spx enhances the degradation of ComK (18), which likely counteracts the overexpression of *comK* and reverses its detrimental effects on cell survival in our system. To examine ComK protein levels in the suppressors, we performed immunoblot analysis. In competence medium, as expected, ComK was present in WT cells and the SigB control before *sigB* expression. Consistent with the idea that upregulating SigB or Spx reduces ComK levels, in the three suppressors (*rsbW* [*D149N*], *sigB++,* or ∆*yjbH*), ComK levels were barely detectable (Fig. 7A left). Next, we tested ComK levels when *comK* expression was induced by *Pspank* and *Pxyl* (a xylose-inducible promoter being used for *Pspank-sigB*) (50). As expected, in exponential growth, ComK was absent in WT cells but present when induced (Fig. 7A, right). Similar to the situation in competence medium, ComK protein levels were barely detectable in *sigB++*, *rsbW^D149N^*, or ∆*yjbH*. These results show that ectopically induced ComK proteins were eliminated in these conditions.

Finally, we analyzed transformation efficiency in the suppressors. In the competence BMK medium, the three suppressors (*rsbW* [*D149N*], *sigB++,* or ∆*yjbH*) all had reduced transformation efficiency compared with the WT (Fig. 7B, left). Specifically, *rsbW* (D149N) reduced competence more than 100-fold, while *sigB* overexpression and ∆*yjbH* reduced transformation frequency to an undetectable level. These results are consistent with previous studies (42, 47) and in line with low ComK protein levels in these cells (Fig. 7A, left). Similarly, when *comK* was ectopically expressed using *Pspank* or *Pxyl* (Fig. 7B, right), all three suppressors (*rsbW* [*D149N*], *sigB++,* or ∆*yjbH*) reduced competence for 10,000-fold or greater, which is consistent with the low ComK protein levels seen by immunoblotting (Fig. 7A).

Overall, our results show that in *Pspank*- or *Pxyl-comK*, although the suppressor mutations allow cells to survive with normal replication and cell division (Fig. 5 and 6), they in turn reduce transformation efficiency (Fig. 7B). Thus, competence cannot be uncoupled from the inhibition of normal cellular activities.

## DISCUSSION

**Fig 8:**
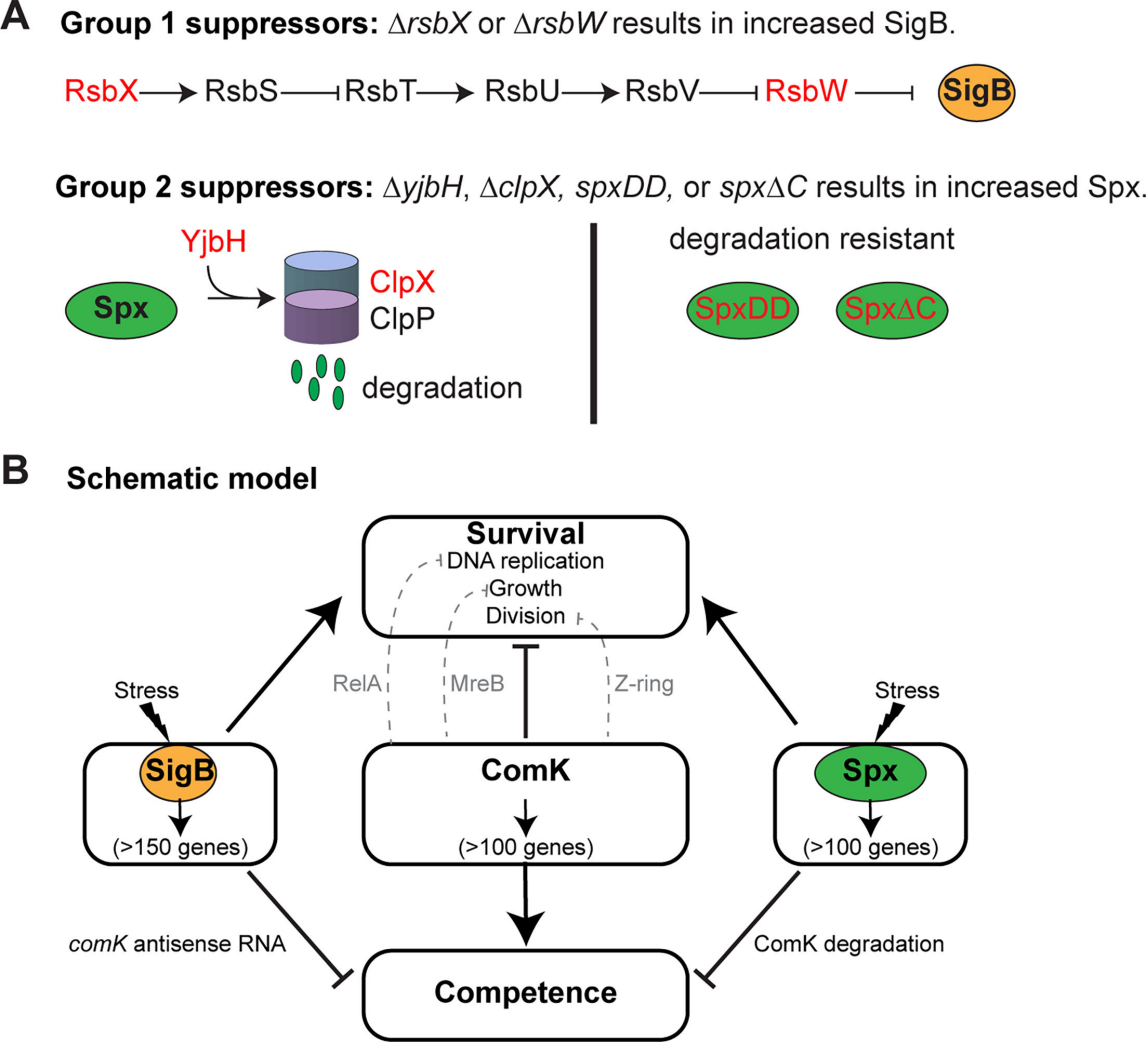
Schematic model. (**A**) Mutants that suppressed *comK*-induced cell death result in increased protein levels of SigB or Spx. Group 1 suppressors are related to the regulation of SigB. Group 2 suppressors are related to the protein level of Spx. Red color indicates proteins that are mutated. (**B**) The model presenting the effects of ComK, SigB, and Spx on competence and cell survival.

In *B. subtilis*, competence is normally induced during the stationary phase of cell growth. Transient overexpression of *comK* during exponential phase growth resulted in high levels of competence, but prolonged overexpression of *comK* induced cell death through inhibition of DNA replication and cell division. To identify genes that could suppress ComK-induced cell death, we used both Tn-seq and spontaneous suppressor screens. We showed that mutations resulting in increased expression of either the SigB regulon or the Spx regulon restored replication and cell growth (Fig. 8A). However, increasing expression of these regulons also caused loss of competence, negating the idea of keeping *comK*-expressing cells healthy to increase transformation efficiency. These results open interesting discussions about how and why cell division and replication are inhibited during competence, and how and why *sigB* or *spx* upregulation reduces competence.

Growth arrest is an intrinsic consequence of competent cells. A ComK-induced protein, ComGA, contributes to this process by inhibiting elongation, cell division, and DNA replication (30, 51, 52) (Fig. 8B). ComGA is a membrane-associated ATPase involved in the uptake of DNA during competence (52). Evidence suggests that ComGA sequesters MreB to prevent cell elongation (53), inhibits FtsZ-ring formation to block cell division (51), and interacts with RelA to increase (p)ppGPP level, thereby inhibiting DNA replication during competence (30). In addition to ComGA, ComK-induced protein, Maf, has been shown to inhibit cell division through an unknown mechanism (54). Our Tn-seq did not identify *comGA* or *maf* as suppressors (Fig. S2), indicating that transposon insertions in *comGA* or *maf* alone were not able to suppress ComK-induced cell death. These results support the idea that multiple factors inhibit essential cellular processes in competent cells.

Why does natural competence need to be coupled with inhibition of replication and cell division? Blocking replication and cell division during competence would give cells time to repair DNA nicks and gaps generated by recombination prior to beginning a new round of replication to preserve genome integrity (55). In addition, inhibition of cell growth and division allows cells to tolerate antibiotics (30). Thus, temporary blockage of replication, growth, and cell division can be beneficial to cells that are actively uptaking DNA and undergoing recombination. However, our results show that *comK*-expressing cells are inhibited for DNA replication and cell division in the absence of exogenous DNA. Thus, these cellular activities are inhibited on the off chance that DNA will be taken up. This paradox might explain why the natural population is in a bistable state in which only ~10% of the cells are competent while the rest of the population is not (10, 23–25).

Our work revealed that upregulating the *sigB* or *spx* pathways during *comK* overexpression restored replication, cell division, and viability but reduced competence by lowering ComK levels (Fig. 7 and 8B). SigB is an alternative sigma factor that is upregulated by a wide range of stresses and shocks, including temperature, acid, salt, antibiotics, nutrient limitation, energy depletion, and more (33, 34). SigB induces the expression of over 150 genes to deal with these stresses. It was recently reported that SigB induces the expression of antisense RNA of *comK* which reduces the translation of ComK and inhibits competence (47). Our result showing low ComK protein level in *sigB++* is consistent with the idea that the antisense RNA inhibits *comK* translation both *in cis* and *in trans* (47), and that SigB reverses *comK*-induced cell death by preventing the production of ComK. Spx suppresses *comK-*induced cell death in a similar way. *In vitro* evidence indicates that Spx enhances the degradation of ComK (18), which aligns with our results demonstrating low ComK levels when Spx was upregulated in the ∆*yjbH* strain (Fig. 7A, last lane). Spx is shown to increase the expression of ZapA, stabilizing the FtsZ ring and enhancing cell division, which could also reverse the cell division caused by ComK overexpression in our system (Fig. S1) (56, 57).

Our work reinforces the notion that stress responses intrinsically inhibit competence (47). Previous work showed that stress responses inhibit sporulation, which is another developmental process for cells in stationary phase (47, 58–60). These observations indicate that under stresses, cells concentrate their energy toward survival strategies, such as DNA replication and cell division, while inhibiting costly differentiation programs such as sporulation and competence.

In summary, we show that overexpression of *comK* inhibits DNA replication and cell division, ultimately leading to cell death. However, upregulating the SigB or Spx regulon rescued cell death by degrading ComK, abolishing competence. Although we were unsuccessful in increasing transformation efficiency, our findings underscore the intricate interplay between cell growth, competence, and stress responses.

## MATERIALS AND METHODS

### General methods

*B. subtilis* strains were derived from the prototrophic strain PY79 (61). Strains, plasmids, oligonucleotides, and next-generation sequencing samples used in this study are listed in Tables S1 through S4. Cells were grown in defined rich casein hydrolysate (CH) medium (62) at 37°C. Inducers are used at the following concentrations: 1 mM IPTG (Dot Scientific, DS102125) for liquid medium, 0.5 mM IPTG for solid medium, and 0.5% xylose (VWR, IC10330090) for both liquid and solid media. Antibiotics were used at the following final concentrations: phleomycin (Dot Scientific, DSP20200-0.025) 0.8 µg/mL, chloramphenicol (VWR, 0230-100G) 5 µg/mL, kanamycin (VWR, 0408-100G) 10 µg/mL, spectinomycin (Dot Scientific, DSS23000-25) 100 µg/mL, and erythromycin [Dot Scientific, DSE57000-50] 1 µg/mL plus lincomycin [c, J61251] 25 µg/mL (MLS). Cultures were diluted in warm medium so that the cells were in mid-exponential growth with OD_600_ below 0.6 at all time points.

### Fluorescence microscopy

Fluorescence microscopy was performed using a Nikon Ti2 microscope equipped with Plan Apo 100×/1.45NA phase contrast oil objective and an sCMOS camera. Membranes were stained with FM4-64 (*N*-(3-triethylammoniumpropyl)−4-(6-(4-(diethylamino) phenyl) hexatrienyl) pyridinium dibromide, Fisher T-3166) at 3 µg/mL. DNA was stained with DAPI (4´,6-diamidino-2-phenylindole, Fisher D1306) at 2 µg/mL. Images were captured using Nikon Elements 5.11 software and cropped and adjusted using MetaMorph 7.7 software.

### Spontaneous suppressors

Spontaneous suppressors were selected using a *comK*-expressing strain BWX4518, which has two copies of IPTG-inducible *comK* inserted at different genetic locations to reduce the false-positive hits that reside in the *lacO/lacI* expression control system, and one copy of xylose-inducible *comK* for confirmation purposes. The cells were grown in liquid culture in the absence of 1 mM IPTG, then spread on plates containing 0.5 mM IPTG. About 1 in 10^5^ cells formed colonies on IPTG plates. For the colonies that grew on IPTG plates, we streaked them on IPTG plates as well as plates containing 0.5% xylose. The isolates that did not grow on xylose plates likely contained mutations in the *lacO/lacI* control system and were omitted for further testing. The colonies that grew on xylose plates were further analyzed by whole genome sequencing. To avoid picking siblings, we performed this experiment in 10 independent liquid cultures and chose only one suppressor per culture for sequencing.

### WGS

Approximately 5 × 10^8^ exponentially growing cells were collected for each WGS sample. Genomic DNA (gDNA) was extracted using Qiagen DNeasy Kit (69504), sonicated using a Qsonica Q800R2 water bath sonicator, prepared using NEBNext UltraII kit (E7645), and sequenced using Illumina NextSeq500. The reads were mapped to the genome of *B. subtilis* PY79 (NCBI reference sequence NC_022898.1) using CLC Genomics Workbench (CLC Bio, QIAGEN) with the following parameters: mismatch cost = 2; insertion cost = 3; deletion cost = 3; length fraction = 0.6; similarity fraction = 0.97. For marker frequency analysis, the sequencing reads were normalized by the total number of reads before mapping as described above. Plotting and analysis were performed using R scripts. *ori:ter* ratios were calculated using the average number of reads within 30 kb of *oriC* and 30 kb of the *dif* site via a Python script.

Specific mutations were identified using the built-in package of CLC Genomics Workbench (CLC Bio, QIAGEN). InDels and structural variants were detected using the following parameters: *P*-value threshold = 0.0001 (default); maximum number of mismatches = 100; minimum number of reads = 4. Basic variations were detected using these parameters: ignore positions with coverage >100,000; ignore broken pairs; ignore non-specific matches; minimum coverage = 2; minimum count = 1; minimum frequency = 50.0; no base quality filters (all data should be high quality); no read direction filter; relative read direction filter; significance = 1.0; no technology-specific filters.

### Tn-seq to identify suppressors

To obtain transposon insertions that suppressed ComK-induced cell death, we introduced a transposon (pWX642) (63) into a *comK*-expressing strain (BWX4512). pWX642 contains a temperature-sensitive origin for *B. subtilis*, an allele of mariner Himar1 transposase, an erythromycin-resistant gene for plasmid selection, and a spectinomycin resistance gene flanked by inverted repeats that can be recognized by the transposase. One of the inverted repeats was engineered to have a MmeI recognition site. The plasmid was transformed into BWX4512, which has two copies of IPTG-inducible *comK* to reduce the chance of the transposon disrupting the induction system. Transformants were selected on erythromycin plates, then inoculated in liquid medium containing spectinomycin and grown at 22°C overnight. The control library was selected on LB plates containing spectinomycin at 42°C. The suppressor library was selected on the spectinomycin plates at the same condition but also contained 0.5 mM IPTG. About 1 in 10^4^ transposants grew in the presence of IPTG. About 1 million colonies were scraped for the control library and about 100,000 for suppressors. Two biological replicates were harvested for each condition.

Five OD_600_ units of cells from each pool were used for gDNA isolation using QIAGEN DNeasy Blood & Tissue Kit (69504). Three micrograms of gDNA was digested with MmeI (NEB R0637S) for 90 min, then treated with quick calf intestinal alkaline phosphatase (CIP) (NEB M0525L) for 60 min at 37°C. The DNA was extracted using phenol-chloroform, precipitated using ethanol, and resuspended in 15 µL ddH_2_O. The digested DNA end was ligated to an annealed adapter (64) using T4 DNA ligase and incubated at 16°C for about 16 hours. Adapter-ligated DNA was amplified with the primers complementary to the adapter and the transposon inverted repeat sequence using 16 cycles. The PCR product was gel-purified and sequenced at the IU Center for Genomics and Bioinformatics using NextSeq500. Sequencing reads were mapped to *B. subtilis* 168 genome (NCBI NC_00964.3) and analyzed using a procedure described previously (32, 64). The results were visualized and analyzed using Artemis (https://www.sanger.ac.uk/tool/artemis/).

### Immunoblot analysis

Cells were collected in indicated growth conditions, and then subsequently resuspended in lysis buffer (20 mM Tris pH 7.0, 1 mM EDTA, 10 mM MgCl_2_, 1 mg/mL lysozyme, 10 µg/mL DNase I, 100 µg/mL RNase A, 1 mM phenylmethylsulfonyl fluoride (PMSF) and 1% proteinase inhibitor cocktail (Sigma P-8340) to a final OD_600_ of 10 for equivalent loading. The cell resuspensions were incubated at 37°C for 10 min for lysozyme treatment and followed by the addition of an equal volume of 2× Laemmli sample buffer (Bio-Rad 1610737) containing 10% β-mercaptoethanol. Samples were heated for 5 min at 95°C prior to loading. Proteins were separated by precast 4-20% polyacrylamide gradient gels (Bio-Rad 4561096), electroblotted onto mini polyvinylidene fluoride (PVDF) membranes using Bio-Rad Transblot Turbo system and reagents (Bio-Rad 1704156). The membranes were blocked in 5% nonfat milk in phosphate-buffered saline (PBS) with 0.5% Tween-20, then probed with anti-ComK (1:1,000) (65) (gift from David Dubnau) or anti-SigA (1:10,000) (66) diluted into 3% bovine serum albumin (BSA) in 1× PBS-0.05% Tween-20. Primary antibodies were detected using Immun-Star horseradish peroxidase-conjugated goat anti-rabbit antibodies (Bio-Rad 1705046) and Western Lightning Plus ECL chemiluminescence reagents, as described by the manufacturer (PerkinElmer NEL1034001). The signal was captured using the ProteinSimple FluorChem R system.

### Transformation efficiency

Cells were transformed with 5 ng of PCR product amplified from BWX2098 using oWX853 and oWX858, which contains a spectinomycin antibiotic marker, and 2–3 kb of homology to chromosomal region surrounding the *hbs* gene. For cells grown in competence medium, one colony was inoculated in 1 mL BMK media (27). Cells were rolled for 4 hours at 37°C before 200 µL of cells was removed and 5 ng DNA was added. Cells were then rolled for 2 hours before being serially diluted and spread on LB plates with or without 100 µg/mL spectinomycin. For cells containing *comK-*expressing alleles, one colony was inoculated in 3 mL of CH medium (62) containing inducer (1 mM IPTG or 0.5% xylose). Cells were rolled for 1 hour at 37°C before adding 5 ng DNA and rolling for 2 more hours. Cells were then serially diluted and spread LB plates with or without 100 µg/mL spectinomycin. CFUs were counted on plates that contained 100–300 colonies. Transformation efficiency was calculated by dividing the CFUs on the plates containing spectinomycin by the CFUs on the LB plates. At least three biological replicates were performed. Each biological replicate contained three technical replicates which were averaged to calculate transformation efficiency.

### Strain constructions

BWX4639 (∆*rsbX loxP-kan-loxP*) was constructed by direct transformation of an isothermal assembly (67) product into PY79. The isothermal reaction contained three PCR fragments: (i) region upstream of the *rsbX* gene (amplified from the PY79 genomic DNA using oWX1952 and oWX1953); (ii) *loxP-kan-loxP* cassette (amplified from pWX470 using universal primers oWX438 and oWX439); and (iii) region downstream of the *rsbX* gene (amplified from PY79 genomic DNA using primers oWX1954 and oWX1955). The transformants were PCR amplified using oWX1956 and oWX1957 to confirm the deletion. pWX470 contains a *loxP-kan-loxP* cassette.

BWX4643 (∆*yjbH loxP-kan-loxP*) was constructed by direct transformation of an isothermal assembly (67) product into PY79. The isothermal reaction contained three PCR fragments: (i) region upstream of the *yjbH* gene (amplified from the PY79 genomic DNA using oWX1958 and oWX1959); (ii) *loxP-kan-loxP* cassette (amplified from pWX470 using universal primers oWX438 and oWX439); and (iii) region downstream of the *yjbH* gene (amplified from PY79 genomic DNA using primers oWX1960 and oWX1961). The transformants were PCR amplified using oWX1962 and oWX1963 to confirm the deletion. pWX470 contains a *loxP-kan-loxP* cassette.

BWX2098 (*hbs* [*loxP-spec-loxP*] [*knock in*]) was constructed by direct transformation of an isothermal assembly (67) product into PY79. The isothermal reaction contained three PCR fragments: (i) region upstream of the *hbs* gene (amplified from the PY79 genomic DNA using oWX853 and oWX856); (ii) *loxP-spec-loxP* cassette (amplified from pWX466 using universal primers oWX438 and oWX439); and (iii) region downstream of the *hbs* gene (amplified from PY79 genomic DNA using primers oWX857 and oWX858). pWX466 contains a *loxP-spec-loxP* cassette. The region of the insertion was amplified using oWX447 and oWX488 before being sequenced with oWX442.

BWX5605 (∆*yjbI loxP-kan-loxP*) was constructed by direct transformation of an isothermal assembly (67) product into PY79. The isothermal reaction contained three PCR fragments: (i) region upstream of the *yjbI* gene (amplified from the PY79 genomic DNA using oWX3266 and oWX3267); (ii) *loxP-kan-loxP* cassette (amplified from pWX470 using universal primers oWX438 and oWX439); and (iii) region downstream of the *yjbI* gene (amplified from PY79 genomic DNA using primers oWX3268 and oWX3269). pWX470 contains a *loxP-kan-loxP* cassette. The *loxP-kan-loxP* cassette was subsequently looped out using a *cre*-expressing plasmid pDR244 (27), which contains a spectinomycin resistance gene and a temperature-sensitive replication origin, generating an in-frame deletion of *yjbI*. The region was sequenced using oWX3270 and oWX3271. The unmarked strain is BWX5612 (∆*yjbI loxP no a.b*.).

BWX5607 (∆*rsbV loxP-kan-loxP*) was constructed by direct transformation of an isothermal assembly(67) product into PY79. The isothermal reaction contained three PCR fragments: (i) region upstream of the *rsbV* gene (amplified from the PY79 genomic DNA using oWX3272 and oWX3273); (ii) *loxP-kan-loxP* cassette (amplified from pWX470 using universal primers oWX438 and oWX439); and (iii) region downstream of the *rsbV* gene (amplified from PY79 genomic DNA using primers oWX3274 and oWX3275). pWX470 contains a *loxP-kan-loxP* cassette.

The *loxP-kan-loxP* cassette was subsequently looped out using a *cre*-expressing plasmid pDR244 (27), which contains a spectinomycin resistance gene and a temperature-sensitive replication origin, generating an in-frame deletion of *rsbV*. The region was sequenced using oWX3276 and oWX3277. The unmarked strain is BWX5625 (*∆rsbV loxP no a.b*.).

BWX5608 (∆*rsbW loxP-kan-loxP*) was constructed by direct transformation of an isothermal assembly (67) product into PY79. The isothermal reaction contained three PCR fragments: (i) region upstream of the *rsbW* gene (amplified from the PY79 genomic DNA using oWX3278 and oWX3279); (ii) *loxP-kan-loxP* cassette (amplified from pWX470 using universal primers oWX438 and oWX439); and (iii) region downstream of the *rsbW* gene (amplified from PY79 genomic DNA using primers oWX3280 and oWX3281). PWX470 contains a *loxP-kan-loxP* cassette. The *loxP-kan-loxP* cassette was subsequently looped out using a *cre*-expressing plasmid pDR244 (27), which contains a spectinomycin resistance gene and a temperature-sensitive replication origin, generating an in-frame deletion of *rsbW*. The region was sequenced using oWX3282 and oWX3283. The unmarked strain is BWX5614 (∆*rsbW loxP no a.b*.).

BWX4747 (*rsbW* [*Y59C*]) unmarked mutation was introduced by allelic replacement using single crossover integration plasmid pWX793, which is based on a derivative of pMAD (68) called pMiniMAD2 (69). Essentially, pWX793 was transformed to wild-type strain PY79 and selected on MLS plate. Transformants were grown in liquid culture in the absence of MLS selection to allow for “loop-out” of the plasmid. The culture was plated after serial dilution, and individual colonies were screened for loss of MLS resistance. As the resulting MLS sensitive clones can either contain the mutation of interest (in this case, [*Y59C*]) or reverse to the wild-type, the genomic DNA of candidate clones was amplified using oWX1975 and oWX1976, and sequencing using oWX1974.

BWX4749 (*rsbW* [*D125G*]) and BWX4751 (*rsbW* [*D149N*]) were constructed the same way as the (Y59C) mutant using pWX794 and pWX795.

### Plasmid constructions

pWX682 (*yvbJ::Pspank [natRBS] comK [cat]*) was generated by inserting *comK* with its native ribosome-binding site (natRBS) (amplified from the PY79 genome using oWX1220 and oWX1221 and digested using XmaI and SpeI) into pMS040 between XmaI and SpeI. pMS040 (*yvbJ::Pspank [cat]*) is an ectopic integration vector containing an IPTG-inducible promoter (*Pspank*) and chloramphenicol resistance marker and is a gift from David Rudner at Harvard Medical School, Boston. The plasmid was sequenced using oWX486 and oWX487.

pWX779 (*ycgO::Pspank* [*kan*]) was generated by inserting *kan* resistance marker (liberated from pMS034 using BamHI and SalI) into pER065 (70) between BamHI and SalI. pMS034 (*yhdG::Pspank* [*kan*]) is an ectopic integration vector containing an IPTG-inducible promoter (*Pspank*) and kanamycin resistance marker and is a gift from David Rudner. pER065 (*ycgO::Pspank* [*erm*]) (70) is an ectopic integration vector containing an IPTG-inducible promoter (*Pspank*) and erythromycin resistance marker.

pWX780 (*yhdG::Pspank* [*natRBS*] *comK* [*phleo*]) was generated by inserting *comK* with its natRBS (liberated from pWX682 using HindIII and SpeI) into pMS026 between HindIII and SpeI. pMS026 (*yhdG::Pspank* [*phleo*]) is an ectopic integration vector containing an IPTG-inducible promoter (*Pspank*) and phleomycin resistance marker and is a gift from David Rudner at Harvard Medical School, Boston. The plasmid was sequenced using oWX486 and oWX524.

pWX781 (*ycgO::Pspank* [*natRBS*] *comK* [*kan*]) was generated by inserting *comK* with its natRBS (liberated from pWX682 using XhoI and SpeI) into pWX779 (described above) between XhoI and SpeI. The plasmid was sequenced using oWX486 and oWX524.

pWX787 (*yvbJ::Pxyl [natRBS] comK [cat]*) was generated by inserting *comK* with its natRBS (liberated from pWX682 using HindIII and NheI) into pMS039 between HindIII and NheI. pMS039 (*yvbJ::Pxyl [cat]*) is an ectopic integration vector containing a xylose inducible promoter (*Pxyl*) and chloramphenicol resistance marker and is a gift from David Rudner at Harvard Medical School, Boston. The plasmid was sequenced using oML77 and oWX1894.

pWX792 (*amyE::PgsiB-yfp* [*spec*]) was generated by three-way ligation to insert *gsiB* promoter (*PgsiB*) (amplified from PY79 genome using primers oWX1949 and oWX1950 and digested with HindIII and XhoI) and *yfp* (amplified from BWX1771 [71] using primers oWX1218 and oWX1219 and digested with XhoI and BamHI) into pLD30 (*amyE::spec*) (72) between HindIII and BamHI. The plasmid was sequenced using oML79 and oWX1951.

pWX793 (*pMiniMAD2 rsbW* [*Y59C*] [*erm*]) was generated by isothermal assembly using three fragments: (i) the pMiniMAD2 plasmid (69) digested with EcoRI and BamHI; (ii) *rsbW (Y59C*) up amplified using oWX1964 and oWX1965; (iii) *rsbW (Y59C*) down amplified using oWX1966 and oWX1967. The resulting plasmid makes an unmarked mutation *rsbW* (*Y59C*). The plasmid was sequenced using oWX1974.

pWX794 (*pMiniMAD2 rsbW* [*D125G*] [*erm*]) was generated by isothermal assembly using three fragments: (i) the pMiniMAD2 plasmid (69) digested with EcoRI and BamHI; (ii) *rsbW (D125G*) up amplified using oWX1964 and oWX1968; (iii) *rsbW (D125G*) down amplified using oWX1969 and oWX1970. The resulting plasmid makes an unmarked mutation *rsbW (D125G*). The plasmid was sequenced using oWX1974.

pWX795 (*pMiniMAD2 rsbW* [*D149N*] [*erm*]) was generated by isothermal assembly using three fragments: (i) the pMiniMAD2 plasmid (69) digested with EcoRI and BamHI; (ii) *rsbW (D149N*) up amplified using oWX1964 and oWX1971; (iii) *rsbW (D149N*) down amplified using oWX1972 and oWX1973. The resulting plasmid makes an unmarked mutation *rsbW* (*D149N*). The plasmid was sequenced using oWX1974.

pWX799 (*yhdG::Pspank* [*optRBS*] *sigB* [*erm*]) was generated by inserting *sigB* with an optimized ribosome-binding site (*optRBS*) (amplified from the PY79 genome using oWX1983 and 1984 and digested using SpeI and SphI) into pMS022 between SpeI and SphI. pMS022 (*yhdG::Pspank* [*erm*]) is an ectopic integration vector containing an IPTG-inducible promoter (*Pspank*) and erythromycin resistance marker and is a gift from David Rudner at Harvard Medical School, Boston. The plasmid was sequenced using oWX486 and oWX524.

pWX802 (*yhdG::Phyperspank [optRBS] spx [kan]*) was generated by inserting *spx* with an optimized ribosome-binding site (optRBS) (amplified from the PY79 genome using oWX1985 and 1986 and digested using SpeI and SphI) into pMS036 between SpeI and SphI. pMS036 (*yhdG::Phyperspank [kan]*) is an ectopic integration vector containing an IPTG-inducible promoter (*Phyperspank*) and kanamycin resistance marker and is a gift from David Rudner at Harvard Medical School, Boston. The plasmid was sequenced using oWX486 and oWX524.

pWX804 (*yhdG::Phyperspank [optRBS] spxDD [kan]*) was generated by inserting *spxDD* with an optRBS (amplified from the PY79 genome using oWX1985 and 2005 and digested using SpeI and SphI) into pMS036 between SpeI and SphI. *spxDD* (45) encodes proteinase-resistant SpxDD in which the last two residues of Spx (A130 and N131) are replaced with two Asp residues. pMS036 (*yhdG::Phyperspank [kan]*) is an ectopic integration vector containing an IPTG-inducible promoter (Phyperspank) and kanamycin resistance marker and is a gift from David Rudner at Harvard Medical School, Boston. The plasmid was sequenced using oWX486 and oWX524.

pWX805 (*yhdG::Phyperspank [optRBS] spx∆C [kan]*) was generated by inserting *spx∆C* with an optRBS (amplified from the PY79 genome using oWX1985 and oWX2006 and digested using SpeI and SphI) into pMS036 between SpeI and SphI. *spx∆C* (46) encodes a truncated Spx∆C protein that lacks the final 12 amino acids at the carboxyl terminal of Spx. pMS036 (*yhdG::Phyperspank [kan]*) is an ectopic integration vector containing an IPTG-inducible promoter (*Phyperspank*) and kanamycin resistance marker and is a gift from David Rudner at Harvard Medical School, Boston. The plasmid was sequenced using oWX486 and oWX524.

## Data Availability

High-throughput sequencing results were deposited to the NCBI Sequence Read Archive (SRA) (accession no. PRJNA1256430).

## SUPPLEMENTAL MATERIAL

Supplemental figures and tables spectrum.01612-25-s0001.pdf Fig. S1 and S2, and Tables S1 to S4.

ASM does not own the copyrights to Supplemental Material that may be linked to, or accessed through, an article. The authors have granted ASM a non-exclusive, world-wide license to publish the Supplemental Material files. Please contact the corresponding author directly for reuse.
