# Supplementary material for: ComK-induced cell death is reversed by upregulating the SigB or Spx pathway in Bacillus subtilis
Source: Microbiol Spectr. 2025 Aug 7;13(9):e01612-25. doi: 10.1128/spectrum.01612-25 (PMC12403567; doi:10.1128/spectrum.01612-25)
Supplement: Supplemental figures and tables — Fig. S1 and S2, and Tables S1 to S4. [file spectrum.01612-25-s0001.pdf]

Supplemental Information For

ComK-induced cell death is reversed by upregulating the SigB or Spx pathway in *Bacillus subtilis*

Emma E. Wiesler, Qin Liao, Zhongqing Ren, Kathy F. Zhang, Jin Dai, Yinuo Ma, Gail G. Hardy, and Xindan Wang

Figure S1

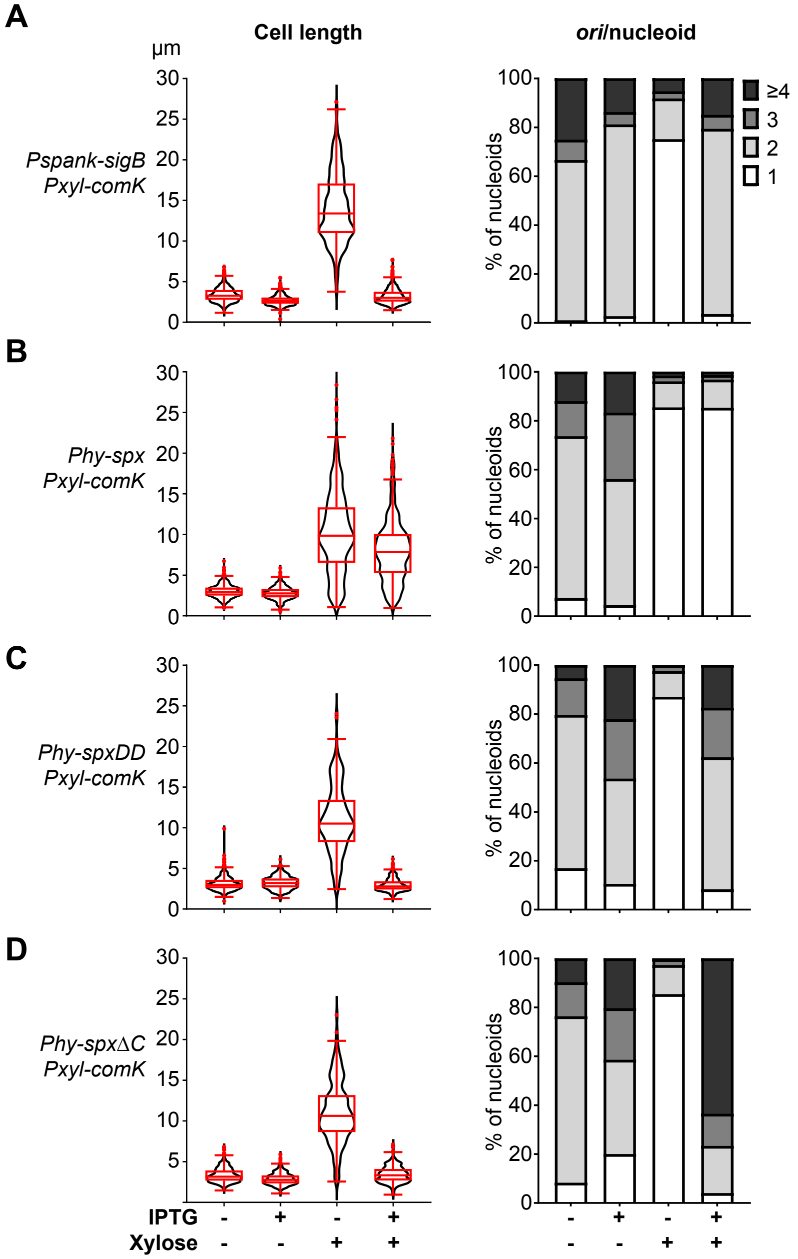
A
B
C
D
Cell length
ori/nucleoid
µm
Pspank-sigB
Pxyl-comK
Phy-spx
Pxyl-comK
Phy-spxDD
Pxyl-comK
Phy-spxΔC
Pxyl-comK
% of nucleoids
≥4
3
2
1
IPTG
Xylose

Figure S1. Quantitative analysis of SigB or Spx expressing cells.

(A-D) Left panels: quantitative analysis of cell length distribution in **Figures 6B-E** (BWX4809, BWX4868, BWX4866, BWX4867). Boxplots show the mean, quartiles, 5th and 95th percentiles of the data. Right panels: quantitative analysis of number of origin per nucleoid in **Figures 6B-E**.

Figure S2

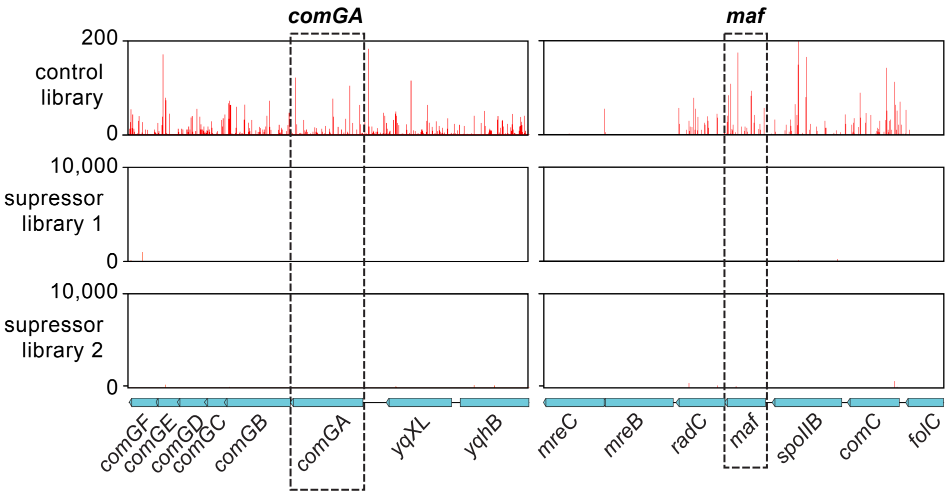
comGA
maf
200
control
library
0
10,000
supressor
library 1
0
10,000
supressor
library 2
0
comGF
comGE
comGD
comGC
comGB
comGA
yqXL
yqhB
mreC
mreB
radC
maf
spoIIB
comC
folC

Figure S2. Transposon insertions in *comGA* and *maf*.

Tn-seq plots showing transposon insertions without *comK* expression (top) or with *comK* expression (bottom, two biological replicates). The x-axis indicates gene locus. The y-axis indicates the number of sequencing reads at each insertion site. Black dotted rectangles highlight regions of interest. Transposon insertions in *comGA* or *maf* alone did not allow the *comK*-expressing cells to survive.

Table S1. Bacterial strains used in this study.

Strain	Genotype	Reference	Figure
**Strains used in main figures**			
PY79	Wild-type		1, 2A, 5AB, 6A, 7
BWX4497	*yhdG::Pspank (natRBS) comK (phleo)*	this study	1, 2A, 7
BWX3253	*yvbJ::Pspank (natRBS) comK (cat), ΔcomK (loxP-erm-loxP)*	this study	1, 2A
BWX3145	*ΔcomK (loxP-erm-loxP)*	this study	1B, 7A
BWX4516	*yycR::tetO48 (erm), ycgO::PftsW tetR-cfp (phleo), yvbJ::Pspank (natRBS) comK (cat)*	this study	2A-E, 5C-E
BWX3174	*yvbJ::Pspank (natRBS) comK (cat)*	this study	2F
BWX4518	*yhdG::Pspank (natRBS) comK (phleo), ycgO::Pspank (natRBS) comK (kan), yvbJ::Pxyl (natRBS) comK (cat)*	this study	3
BWX4512	*yhdG::Pspank (natRBS) comK (phleo), ycgO::Pspank (natRBS) comK (kan)*	this study	4, S2
BWX4661	*yhdG::Pspank (natRBS) comK (phleo), amyE::gsiB-yfp (spec)*	this study	5AB
BWX4699	*yhdG::Pspank (natRBS) comK (phleo), amyE::gsiB-yfp (spec), ΔrsbX (loxP-kan-loxP)*	this study	5A
BWX4700	*yhdG::Pspank (natRBS) comK (phleo), amyE::gsiB-yfp (spec), ΔyjbH (loxP-kan-loxP)*	this study	5A
BWX4759	*rsbW (Y59C) unmarked mutation, yvbJ::Pspank (natRBS) comK (cat), amyE::gsiB-yfp (spec)*	this study	5A
BWX4760	*rsbW (D125G) unmarked mutation, yvbJ::Pspank (natRBS) comK (cat), amyE::gsiB-yfp (spec)*	this study	5A
BWX4761	*rsbW (D149N) unmarked mutation, yvbJ::Pspank (natRBS) comK (cat), amyE::gsiB-yfp (spec)*	this study	5A
BWX4825	*yhdG::Pspank (natRBS) comK (phleo), clpX::kan*	this study	5B
BWX5627	*ΔrsbV (loxP no a.b.), yvbJ::Pspank (natRBS) comK (cat)*	this study	5B
BWX5620	*ΔrsbW (loxP no a.b.), yvbJ::Pspank (natRBS) comK (cat)*	this study	5B
BWX5618	*ΔyjbI (loxP no a.b.), yvbJ::Pspank (natRBS) comK (cat)*	this study	5B

BWX4695	*yycR::tetO48 (erm), ycgO::PftsW tetR-cfp (phleo), yvbJ::Pspank (natRBS) comK (cat), ΔrsbX (loxP-kan-loxP)*	this study	5C-E
BWX4696	*yycR::tetO48 (erm), ycgO::PftsW tetR-cfp (phleo), yvbJ::Pspank (natRBS) comK (cat), ΔyjbH (loxP-kan-loxP)*	this study	5C-E, 7
BWX4766	*rsbW (D125G) unmarked mutation, yvbJ::Pspank (natRBS) comK (cat), yycR::tetO48 (erm), ycgO::PftsW tetR-cfp (phleo)*	this study	5C-E
BWX4767	*rsbW (D149N) unmarked mutation, yvbJ::Pspank (natRBS) comK (cat), yycR::tetO48 (erm), ycgO::PftsW tetR-cfp (phleo)*	this study	5C-E
BWX4827	*yycR::tetO48 (erm), ycgO::PftsW tetR-cfp phleo, yvbJ::Pspank (natRBS) comK (cat), clpX::TnYLB (kan) (TATACACAGCA)*	this study	5C-E
BWX4788	*yycR::tetO48 (phleo), ycgO::PftsW tetR-cfp (spec), yvbJ::Pxyl (natRBS) comK (cat)*	this study	6A
BWX4809	*yycR::tetO48 (phleo), ycgO::PftsW tetR-cfp (spec), yvbJ::Pxyl (natRBS) comK (cat,) yhdG::Pspank (optRBS) sigB (erm)*	this study	6AB, S1A
BWX4868	*yycR::tetO48 (phleo), ycgO::PftsW tetR-cfp (spec), yvbJ::Pxyl (natRBS) comK (cat), yhdG::Phyperspank (optRBS) spx (kan)*	this study	6AB, S1B
BWX4866	*yycR::tetO48 (phleo), ycgO::PftsW tetR-cfp (spec), yvbJ::Pxyl (natRBS) comK (cat), yhdG::Phyperspank (optRBS) spxDD (kan)*	this study	6AB, S1C
BWX4867	*yycR::tetO48 (phleo), ycgO::PftsW tetR-cfp (spec), yvbJ::Pxyl (natRBS) comK (cat), yhdG::Phyperspank (optRBS) spxΔC (kan)*	this study	6AB, S1D
BWX4795	*yhdG::Pspank (optRBS) sigB (erm)*	this study	7
BWX4751	*rsbW (D149N) unmarked mutation*	this study	7
BWX4643	*ΔyjbH (loxP-kan-loxP)*	this study	7
BWX4497	*yhdG::Pspank (natRBS) comK (phleo)*	this study	7
BWX4513	*yhdG::Pxyl (natRBS) comK (phleo)*	this study	7
BWX5716	*yhdG::Pxyl (natRBS) comK (phleo), yhdG::Pspank (optRBS) sigB (erm)*	this study	7
BWX4755	*rsbW (D149N) unmarked mutation, yvbJ::Pspank (natRBS) comK (cat)*	this study	7
**Strains used for strain building**			
BKO1257	Bs168, *trpC2, ΔcomK (loxP-erm-loxP)*	(1)	

DK5580	3610, Δ*clpX (kan)*	Kearns lab	
DS2231	3610, Δ*clpX TnYLB (kan)* (TATACACAGCA)	Kearns lab	
BWX1771	*yycR::tetO48 (cat), ycgO::PftsW tetR-cfp (phleo), lacA::PxylA (Ec) sspB (loxP no a.b.), smc-ssrA (loxP-kan-loxP), dnaX-yfp (spec)*	(2)	
BWX2098	*hbs (loxP-spec-loxP) (knock in)*	this study	
BWX2801	*yycR::tetO48 (erm), ycgO::PftsW tetR-cfp (phleo)*	this study	
BWX4639	Δ*rsbX (loxP-kan-loxP)*	this study	
BWX4747	*rsbW (Y59C)* unmarked mutation	this study	
BWX4749	*rsbW (D125G)* unmarked mutation	this study	
BWX5605	Δ*yjbI (loxP-kan-loxP)*	this study	
BWX5607	Δ*rsbV (loxP-kan-loxP)*	this study	
BWX5608	Δ*rsbW (loxP-kan-loxP)*	this study	
BWX5612	Δ*yjbI (loxP no a.b.)*	this study	
BWX5614	Δ*rsbW (loxP no a.b.)*	this study	
BWX5625	Δ*rsbV (loxP no a.b.)*	this study	

Table S2. Plasmids used in this study.

Plasmid	Description	Reference
pDR244	*cre* recombinase under constitutive expression, temperature-sensitive origin *(spec)*	(1)
pER065	*ycgO::Pspank (erm)*	(3)
pLD30	*amyE::spec*	(4)
pMiniMAD2	*loop-in loop-out vector* that does not have *lacZ*	(5, 6)
pMS022	*yhdG::Pspank (erm)*	D. Z. Rudner unpublished
pMS026	*yhdG::Pspank (phleo)*	D. Z. Rudner unpublished
pMS034	*yhdG::Pspank (kan)*	D. Z. Rudner unpublished
pMS036	*yhdG::Phyperspank (kan)*	D. Z. Rudner unpublished
pMS039	*yvbJ::PxylA (cat)*	D. Z. Rudner unpublished
pMS040	*yvbJ::Pspank (cat)*	D. Z. Rudner unpublished
pWX466	*loxP-spec-loxP*	this study
pWX470	*loxP-kan-loxP*	this study
pWX642	*pACYC TnKRM (spec) (amp) MmeI modified*	(7)
pWX682	*yvbJ::Pspank (natRBS) comK (cat)*	this study
pWX779	*ycgO::Pspank (kan)*	this study
pWX780	*yhdG::Pspank (natRBS) comK (phleo)*	this study
pWX781	*ycgO::Pspank (natRBS) comK (kan)*	this study
pWX787	*yvbJ::Pxyl (natRBS) comK (cat)*	this study
pWX792	*amyE::PgsiB-yfp (spec)*	this study
pWX793	*pMiniMAD2 rsbW (Y59C) (erm)*	this study
pWX794	*pMiniMAD2 rsbW (D125G) (erm)*	this study
pWX795	*pMiniMAD2 rsbW (D149N) (erm)*	this study
pWX799	*yhdG::Pspank (optRBS) sigB (erm)*	this study
pWX802	*yhdG::Phyperspank (optRBS) spx (kan)*	this study
pWX804	*yhdG::Phyperspank (optRBS) spxDD (kan)*	this study

pWX805	*yhdG::Phyperspank (optRBS) spxΔC (kan)*	this study

Table S3. Oligonucleotides used in this study.

Oligos	Sequence	Use
oML077	gttgaactaatgggtgc	sequencing
oML079	ctcttgccagtcacgttacg	sequencing
oWX438	gaccagggagcactggtcaac	universal
oWX439	tccttctgctccctcgctcag	universal
oWX442	ccttgacgagcaagggattgacgc	sequencing
oWX447	gcgctttgcaacggcttcaacggc	sequencing
oWX486	gccgctctagctaagcagaaggc	sequencing
oWX487	aacggtctgataagagacaccggc	sequencing
oWX488	gagtgcttcatctggttacgatc	sequencing
oWX524	ggtacgtacgatctttcagccgactc	sequencing
oWX853	ctatcaatacgtgcttgttgacgtag	BWX2098, transformation assay
oWX856	ctgagcgagggagcagaaggatccgcatacacgatctatattcacaatta	BWX2098
oWX857	gttgaccagtgctccctggtcatctagcttacatacactttatttcttcac	BWX2098
oWX858	gaaatccaagcccttgatctcgccgc	BWX2098, transformation assay
oWX1218	gcgctcgagggttccggaagtaaaggagaagaacttttcac	pWX792
oWX1219	cgcggatccttatttgtatagttcatccatgccatg	pWX792
oWX1220	atacccgggttgcagaaaaaggatggaggcc	pWX682
oWX1221	cgcactagttatgtgacatctcaggtatatggc	pWX682
oWX1894	acatagtacatagcgaatcttccc	sequencing
oWX1949	cgcaagctttgatgttgtcggcaaaagatcg	pWX792
oWX1950	gcgctcgaggctcattttgttattgtctgccat	pWX792
oWX1951	aaactggtctgatcgaaatagtac	sequencing
oWX1952	gttgttgaacaaaacgttgatgcc	BWX4639
oWX1953	ctgagcgagggagcagaaggacttcaacctggatcattacattaactc	BWX4639
oWX1954	gttgaccagtgctccctggtctaaaaaaccagaaaaagaagctggac	BWX4639
oWX1955	cggctcgtctgagatttgttccgc	BWX4639
oWX1956	attgaagcggattcggacggaagc	sequencing
oWX1957	cgcccgatgtttctccacatgctc	sequencing
oWX1958	tcaaaaacatagcatcggcactcc	BWX4643
oWX1959	ctgagcgagggagcagaaggacggttttttggatgaccgtggcaatg	BWX4643
oWX1960	gttgaccagtgctccctggtctagccgcaggcgtgcatatgcttg	BWX4643
oWX1961	tacacaggccgcaagcgcgacagc	BWX4643

oWX1962	ctcagtatttaggcgggcctcctc	sequencing
oWX1963	tagacatcatggcggtcctcctcg	sequencing
oWX1964	ttgtaaaacgacggccagtgaattcgacggcgtcacagaatgcagaacg	pWX793, pWX794, pWX795
oWX1965	cacttccccatttttatcttctttgcaagcgtgctgaaccgcatttgtg	pWX793
oWX1966	cacaaatgcggttcagcacgcttgcaaagaagataaaaatggggaagtg	pWX793
oWX1967	ctgcaggtcgactctagaggatccgcttgatagcttttgcccatttcc	pWX793
oWX1968	ggagtggttttgcactctgacttcgcccatgagcgtttccattaaatatagac	pWX794
oWX1969	gtctatatttaatggaaacgctcatgggcgaagtcagagtgcaaaaccact cc	pWX794
oWX1970	ctgcaggtcgactctagaggatccactgtcaccgcaaatcgatttccc	pWX794
oWX1971	gtttttgatggttgtgtcatgattaactcgctccccatttaaatac	pWX795
oWX1972	gtatttaaatggggagcgagttaatcatgacacaaccatcaaaaac	pWX795
oWX1973	ctgcaggtcgactctagaggatcccctgattacagcgttcgataatgc	pWX795
oWX1974	ctttctgaacggctgatccgactg	sequencing
oWX1975	gacggcgtcacagaatgcagaacg	BWX4747, BWX4749, BWX4751
oWX1976	cctgattacagcgttcgataatgc	BWX4747, BWX4749, BWX4751
oWX1983	cgcactagtacataaggaggaactactatgacacaaccatcaaaaacta cg	pWX799
oWX1984	tttgcatgcttacattaactccatcgagggatcttc	pWX799
oWX1985	cgcactagtacataaggaggaactactatggttacactatacacatcacca agc	pWX802, pWX804, pWX805
oWX1986	tttgcatgcttagtttgccaaacgctgtgcttctc	pWX802
oWX2005	tttgcatgcttagtcatccaaacgctgtgcttctcttaattg	pWX804
oWX2006	tttgcatgcttagcgaacttttcttggcaggaaacg	pWX805
oWX3266	ttgacgcgtccggtcacaaattcg	BWX5605
oWX3267	ctgagcgagggagcagaaggagcgttaaacgattgtcccatgttg	BWX5605
oWX3268	gttgaccagtgctccctggtcaatcaaacggaagcggaggatcg	BWX5605
oWX3269	tcaggagcttacggcacgaatccg	BWX5605
oWX3270	gcattcctcctttccaagaaacgc	sequencing
oWX3271	aaagcggctgatcctgttcgaacc	sequencing
oWX3272	gtgtccctgtcgtcgatacgatgg	BWX5607
oWX3273	ctgagcgagggagcagaaggatttatattcattcgtatcacctc	BWX5607
oWX3274	gttgaccagtgctccctggtctcagaaggtggagtgcaatgaag	BWX5607

oWX3275	cgcccgatgtttctccacatgc	BWX5607
oWX3276	ggactcgttctcggcatctcgc	sequencing
oWX3277	tgtacggccctagatcctgctgc	sequencing

Table S4. Next Generation Sequencing samples used in this study.

Sample	Biosample accession
WGS_BWX3174_0h	SAMN48179491
WGS_BWX3174_IPTG_1h	SAMN48179492
WGS_BWX3174_IPTG_2h	SAMN48179493
WGS_BWX3174_IPTG_3h	SAMN48179494
WGS_sok_parent_BWX4518	SAMN48179495
WGS_sok01	SAMN48179496
WGS_sok02	SAMN48179497
WGS_sok03	SAMN48179498
WGS_sok04	SAMN48179499
WGS_sok05	SAMN48179500
WGS_sok06	SAMN48179501
WGS_sok07	SAMN48179502
WGS_sok08	SAMN48179503
WGS_sok09	SAMN48179504
WGS_sok10	SAMN48179505

References

1. Koo BM, Kritikos G, Farelli JD, Todor H, Tong K, Kimsey H, Wapinski I, Galardini M, Cabal A, Peters JM, Hachmann AB, Rudner DZ, Allen KN, Typas A, Gross CA. 2017. Construction and Analysis of Two Genome-Scale Deletion Libraries for Bacillus subtilis. Cell Syst 4:291-305 e7.
2. Wang X, Tang OW, Riley EP, Rudner DZ. 2014. The SMC condensin complex is required for origin segregation in Bacillus subtilis. Curr Biol 24:287-92.
3. Meisner J, Montero Llopis P, Sham LT, Garner E, Bernhardt TG, Rudner DZ. 2013. FtsEX is required for CwlO peptidoglycan hydrolase activity during cell wall elongation in Bacillus subtilis. Mol Microbiol 89:1069-83.
4. Garsin DA, Paskowitz DM, Duncan L, Losick R. 1998. Evidence for common sites of contact between the antisigma factor SpoIIAB and its partners SpoIIAA and the developmental transcription factor sigmaF in Bacillus subtilis. J Mol Biol 284:557-68.
5. Arnaud M, Chastanet A, Debarbouille M. 2004. New vector for efficient allelic replacement in naturally nontransformable, low-GC-content, gram-positive bacteria. Appl Environ Microbiol 70:6887-91.
6. Patrick JE, Kearns DB. 2008. MinJ (YvjD) is a topological determinant of cell division in Bacillus subtilis. Mol Microbiol 70:1166-79.
7. Dobihal GS, Flores-Kim J, Roney IJ, Wang X, Rudner DZ. 2022. The WalR-WalK signaling pathway modulates the activities of both CwlO and LytE through control of the peptidoglycan deacetylase PdaC in Bacillus subtilis. J Bacteriol 204:e0053321.
